# Role of metabolite transporters in source-sink carbon allocation

**DOI:** 10.3389/fpls.2013.00231

**Published:** 2013-07-02

**Authors:** Frank Ludewig, Ulf-Ingo Flügge

**Affiliations:** Botanical Institute II, Cologne Biocenter, University of Cologne Cologne, Germany

**Keywords:** source–sink, carbon allocation, metabolite transporters, yield, storage

## Abstract

Plants assimilate carbon dioxide during photosynthesis in chloroplasts. Assimilated carbon is subsequently allocated throughout the plant. Generally, two types of organs can be distinguished, mature green source leaves as net photoassimilate exporters, and net importers, the sinks, e.g., roots, flowers, small leaves, and storage organs like tubers. Within these organs, different tissue types developed according to their respective function, and cells of either tissue type are highly compartmentalized. Photoassimilates are allocated to distinct compartments of these tissues in all organs, requiring a set of metabolite transporters mediating this intercompartmental transfer. The general route of photoassimilates can be briefly described as follows. Upon fixation of carbon dioxide in chloroplasts of mesophyll cells, triose phosphates either enter the cytosol for mainly sucrose formation or remain in the stroma to form transiently stored starch which is degraded during the night and enters the cytosol as maltose or glucose to be further metabolized to sucrose. In both cases, sucrose enters the phloem for long distance transport or is transiently stored in the vacuole, or can be degraded to hexoses which also can be stored in the vacuole. In the majority of plant species, sucrose is actively loaded into the phloem via the apoplast. Following long distance transport, it is released into sink organs, where it enters cells as source of carbon and energy. In storage organs, sucrose can be stored, or carbon derived from sucrose can be stored as starch in plastids, or as oil in oil bodies, or – in combination with nitrogen – as protein in protein storage vacuoles and protein bodies. Here, we focus on transport proteins known for either of these steps, and discuss the implications for yield increase in plants upon genetic engineering of respective transporters.

## LIMITATIONS FOR PLANT YIELD AND GENERAL CONSIDERATIONS

Humans often exploit plant organs that serve plants for their persistence. Organs such as seeds, roots, tubers, or fruits contain considerable energy stored as oil, protein, sugar, or starch that allow the plant to start a new life cycle or its offspring to germinate. Seeds frequently are accompanied by or are embedded in fruits which also contribute to reproduction in that animals eat and often spread these fruits. Humans use these nutritious organs as food or feed and thousands of years ago had already started to propagate plants with better qualities by selective breeding. After long phases of selection-, classical and recently SMART (Selection with Markers and Advanced Reproductive Technologies; McCouch, 2004) breeding, the limits of quality or yield enhancement might be almost reached for some crops. In addition to these breeding programs, transgenic plants have been successfully engineered that often outcompete classically bred cultivars, e.g., plants with enhanced resistances against pathogens or herbicides. Although sometimes unacknowledged, especially by the European society, future prospects for transgenic plants being a farmer’s first choice are intact. It can be assumed that new generations of transgenic plants also include those which are genetically engineered in their source- and/or sink capacity. These features are closely linked to the harvest index, i.e., weight of the grain (or other harvestable organs) divided by the total plant weight. Indeed, genetically engineered crop plants with changes in source- and/or sink capacities were created which were either impaired (e.g., Riesmeier et al., 1994) or improved in yield (e.g., Jonik et al., 2012), demonstrating the feasibility and putative impact of such approaches on yield increase.

In general, the yield of a given cultivar is limited by several factors, and yield can only be improved by unrestricting these limitations. The challenge therefore is to know the limiting factor(s) of the respective crop.

This view, however, should be extended by referring to what is known about the cross-talk between source- and sink activities. When carbon reserves of storage organs are used up by the newly emerging seedling, photosynthesis begins and atmospheric CO_2_ is assimilated. Given an otherwise optimal supply with water, nitrogen, and other nutrients, only the rate of CO_2_ fixation determines plant growth during the vegetative phase. During further development toward maturation, storage organs are produced. The total of all sink organs then additionally determines the photosynthetic rate, as decreasing demand of the sink tissues for carbon ultimately limits photosynthesis, a phenomenon known as “sink limitation of photosynthesis” (Stitt, 1991; Paul and Foyer, 2001; Paul and Pellny, 2003; Ainsworth and Bush, 2011). Therefore, retention of the sink tissues to draw photosynthetic carbon would maintain photosynthesis (McCormick et al., 2006, 2008).

Here, we describe the allocation of carbon from source- to sink tissues and focus on the metabolite transporters known to be involved. We will also focus on C3 plants as the majority of these transporters have been described from these plant species.

## ALLOCATION OF CARBON WITHIN SOURCE LEAVES

Production of carbon compounds occurs during photosynthesis in the chloroplasts. When the assimilated carbon exceeds the local need, carbon can then be exported. Net photoassimilate exporting leaves are defined as “source” (Turgeon, 1989). However, assimilated carbon can either be transiently stored as starch in chloroplasts or transported to the cytosol. From there, it can be directly exported from the leaf or transported into and transiently stored in the vacuole. From both compartments, chloroplasts and the vacuole, carbon can be exported when sink demand exceeds the actual production via photosynthesis, e.g., and of course during the diurnal cycle (starch release from the chloroplast at night). Transient storage of assimilated carbon in source leaves during the day could be regarded as a valve for excess carbon production and might be important for maintaining a high photosynthesis rate. At the same time, plants need this transiently stored carbon pool during the night to maintain sucrose formation for metabolic processes.

### CHLOROPLAST TRANSPORTERS

**FIGURE 1:**
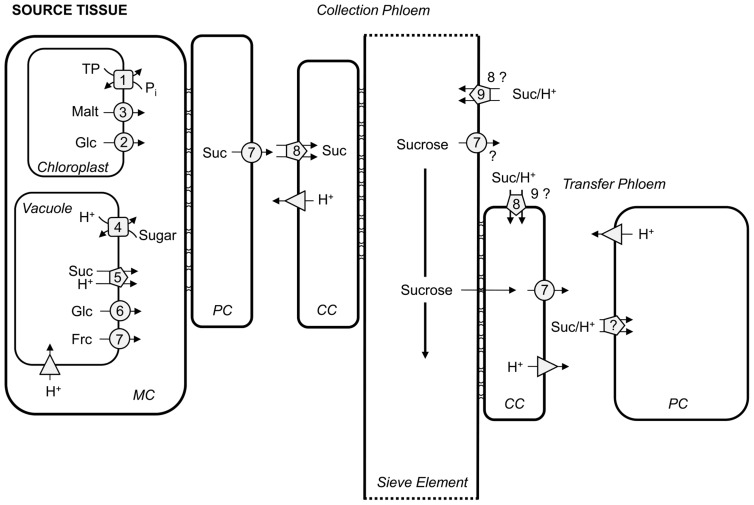
**Overview of carbon transport proteins in source leaves.** Transport steps in mesophyll cells (MC), parenchyma cells (PC), companion cells (CC), and sieve elements are depicted indicating their respective mode of action. Squares represent antiporters, circles describe facilitators, pentagons depict symporters, and triangles H^+^-ATPases/PPases. 1, TPT; 2, pGlcT; 3, MEX; 4, TMT and VGT; 5, SUC4/SUT4; 6, ESL1; 7, SWEETs; 8, SUC2/SUT1; 9, SUC3. TP, triose phosphates; Malt, maltose; Glc, glucose; Suc, sucrose; Frc, fructose. Please refer to text for transporter abbreviations.

During photosynthesis CO_2_ is assimilated in a series of reactions known as the Calvin–Benson cycle. One of six triose phosphates produced can either be used for starch production or can be exported from chloroplasts to the cytosol for sucrose synthesis or respiration. This export is accomplished by the triose phosphate/phosphate translocator (TPT; “1” in **Figure 1;** Fliege et al., 1978; Flügge et al., 1989) which exports triose phosphates in counter-exchange with orthophosphate (day path of carbon). Knock-out and -down *tpt* mutants and transgenic plants produce high amounts of starch during the day which are then partially degraded again during the day (*Arabidopsis*, tobacco; Häusler et al., 1998, 2000; Schneider et al., 2002; Walters et al., 2004; Schmitz et al., 2012) or the following night period (potato; Riesmeier et al., 1993a; Heineke et al., 1994). By this, *tpt* mutants and antisense plants manage to grow normally without any aberrant phenotype. Overexpression of the TPT was performed in tobacco using the *Flaveria trinervia* *TPT* gene (Häusler et al., 2000) and in *Arabidopsis* using the endogenous gene (Cho et al., 2012) with only minor effects. When a cytosolic fructose 1,6-bisphosphatase (cFBPase) was simultaneously overexpressed, *Arabidopsis* plants were larger and exhibited a higher photosynthetic capacity (Cho et al., 2012). Unfortunately, no information on seed yield was provided. Thus, from this approach it cannot be deduced whether *Arabidopsis* yield could possibly be source-limited.

During the night, carbon export from the chloroplast starts with the breakdown of transitory starch and then export of the resulting products maltose and glucose. Weise et al. (2004) found evidence for maltose as the main product exported from chloroplasts at night and proposed a maltose transporter in addition to the plastid glucose transporter (pGlcT; “2” in **Figure 1;** Weber et al., 2000). Export of maltose is mediated by the maltose transporter MEX1 (“3” in **Figure 1;** Niittylä et al., 2004). The loss-of-function “maltose excess” mutant *mex1* was impaired in growth, had a light green yellowish appearance and accumulated maltose and starch. Overexpression of the endogenous transporter (Niittylä et al., 2004) as well as the apple (*Malus domestica*) transporter (Reidel et al., 2008) complemented the mutant phenotype. None of the lines that rescued the *mex1* phenotype displayed any additional aberrant phenotype indicating that *MEX1* overexpression would not lead to increased growth or increased source capacity. Double *mex1*/*tpt-2* or *mex1*/*pglct* but not *pglct*/*tpt-2* mutants displayed an even more severe growth phenotype than *mex1* (Cho et al., 2011) as well as a combination of starch synthesis (*adg1-1*) or degradation (*starch-excess 1-3*, *sex1-3*) mutants with *tpt* mutants (Schneider et al., 2002). The severity of the respective phenotypes differed and the underlying reason is not yet fully understood, however, this might include retrograde signals from the chloroplasts to the nucleus (Stettler et al., 2009;Schmitz et al., 2012).

### VACUOLAR TRANSPORTERS

In source leaves, sugar can be temporarily stored in vacuoles, e.g., when sucrose export via the phloem is saturated (Martinoia et al., 2000). The designation of sugar storage as “temporary” implies the existence of vacuolar sugar importers and exporters. The types of putative sugar importers known to date have been characterized from *Arabidopsis thaliana*, tonoplast monosaccharide transporters (TMTs; “4” in **Figure 1;** Wormit et al., 2006) and vacuolar glucose transporters (VGTs; “4” in **Figure 1;** Aluri and Büttner, 2007). Both types of transporters belong to the monosaccharide transporter (-like) gene family, form a sub-clade each (Büttner, 2007, 2010) and mediate an energy-dependent transport driven by V-type H^+^-ATPases and vacuolar H^+^-PPases. The transport direction by patch clamp technique has been shown to be sugar import into vacuoles for the TMTs (Wingenter et al., 2010; Schulz et al., 2011), while the function of VGT1 in seed germination and flowering as revealed by analyzing vgt1 mutant plants also indicated glucose import into vacuoles (Aluri and Büttner, 2007). TMTs have been shown to import sugar in a proton antiport manner (Wingenter et al., 2010; Schulz et al., 2011), and putative VGT1-mediated glucose import is likely to be also coupled to proton antiport (Aluri and Büttner, 2007). Whereas initially and in accordance with their name TMTs were thought to transport monosaccharides across the tonoplast membrane (Wormit et al., 2006; Wingenter et al., 2010) recently it has been shown that also sucrose is transported by TMTs (Schulz et al., 2011). The *Arabidopsis* genome contains three TMT genes indicating possible redundancy for TMT function. Indeed, when analyzing loss-of-function tmt mutants, only tmt1/tmt2/tmt3 triple or tmt1/tmt2 double knock-out mutants showed impaired uptake of glucose into isolated mesophyll vacuoles (Wormit et al., 2006) or reduced sugar-induced changes in currents in patch clamp analyses (Wingenter et al., 2010), respectively. Double mutants grew worse, compared to wild-type, and were also impaired in seed yield, whereas double mutants additionally overexpressing the *Arabidopsis* TMT1 under the control of the 35S-CaMV promoter “over”-rescued the mutant phenotype due to higher TMT activity. This led to larger plants with increased seed yield. It was hypothesized that sugar sensing is diminished in overexpressing lines due to an increased import of sugars into the vacuole and – more importantly – out of the cytosol. These findings might indicate *Arabidopsis* “yield” to be source-limited (Wingenter et al., 2010). A sink effect, however, cannot be ruled out when using a constitutive promoter for the overexpression of TMT1. One could also imagine a sink effect, in that, sugar is transiently stored in vacuoles of embryo cells to perpetuate sucrose flow to sinks and thus increase sink strength.

Sugar export from the vacuole, on the contrary, is likely to occur in a proton symport manner or, when subcellular concentrations of the sugar to be transported allow for it, can occur by facilitated diffusion. To avoid a futile cycling of sugars into and out of the vacuole and thereby diminishing the proton gradient across the tonoplast, at least one of these processes should be regulated. Indeed, there is evidence for post-translational regulation of TMTs by phosphorylation. A member of the mitogen-activated protein 3-kinase called VH1-interacting kinase (VIK) was found to activate TMTs (Wingenter et al., 2011).

Sucrose transporters of type IV (SUC4/SUT4; “5” in **Figure 1;** Sauer, 2007; Kühn and Grof, 2010; Payyavula et al., 2011) of several species have been found to localize to the vacuolar membrane (Endler et al., 2006; Reinders et al., 2008; Eom et al., 2011; Payyavula et al., 2011; Schneider et al., 2012). The same clade has been designated “type III” by Reinders et al. (2012). Some of these sucrose transporters have been reported to localize to the plasma membrane (Weise et al., 2000; Weschke et al., 2000; Chincinska et al., 2008; Kühn and Grof, 2010). Although still under debate, we only discuss the function of this group of sucrose transporters as vacuolar transporters here. Schulz et al. (2011) were able to measure sucrose-driven proton import into the vacuole under an inverted pH gradient when overexpressing a functional AtSUC4–GFP (green fluorescent protein) construct in the background of the quadruple *tmt1*/*tmt2*/*vgt1*/*vgt2* mutant – to overcome sugar/H^+^-antiport activities – using the patch clamp technique. This result can only be interpreted as sucrose/H^+^ co-export from vacuoles mediated by SUC4 *in vivo*. An *Arabidopsis* *suc4* loss-of-function mutant did not display any aberrant phenotype whereas constitutive SUC4 overexpressed seedlings had slightly less sucrose than wild-type (Schneider et al., 2012). The possible impact of SUC4 under- or overexpressing plants on seed yield was not reported by the authors. In contrast, *sut2* mutants of rice (OsSUT2 belonging to clade IV) had a reduced sugar export ability and accumulated sugars in source leaves which led to several aberrant phenotypes, among them growth retardation, reduction in tiller number and decreased grain weight (Eom et al., 2011). These findings are in line with the interpretation that SUT2-mediated export of sucrose from vacuoles is important to sustain source capacity in rice. It remains to be elucidated whether or not the overexpression of SUT2 in rice leads to increased grain yield.

Evidence for a tonoplast hexose exporter has been gathered by Yamada et al. (2010). The authors mistargeted a slightly mutagenized *Arabidopsis* ESL1, a tonoplast-residing member of the ERD6-like monosaccharide transporters (“6” in **Figure 1;** Büttner, 2007, 2010), to the plasma membrane of tobacco BY-2 cells. Glucose transport across the membrane could be measured using ^14^C-glucose. Addition of high concentrations of unlabeled hexoses inhibited ^14^C-glucose transport to different extents depending on the sugar used for inhibition. The transport was found to be independent of a proton gradient, and determination of the *K*_ m_ for glucose revealed ESL to be a low affinity facilitator exporting monosaccharides out of the vacuole (Yamada et al., 2010). *ESL1* is expressed upon drought and salt stress, as are vacuolar invertases that produce hexoses from sucrose. ESL1-mediated export of hexoses from vacuoles might contribute to the plant’s attempt to increase the osmotic potential within cells. The *esl1* mutant, however, did not display any aberrant phenotype, perhaps due to redundancy (Yamada et al., 2010).

Another *Arabidopsis* ERD6-like monosaccharide transporter, ERDL6, has been described as vacuolar glucose exporter by Poschet et al. (2011). Expression of *ERDL6* is induced when sugars are mobilized, e.g., in darkness, upon heat stress or wounding, and is reduced upon cold stress or external feeding of sugars to the plant. Elevated levels of glucose, but not of fructose and sucrose, were found in *erdl6* mutants, and non-aqueous fractionation revealed a more pronounced glucose increase in vacuoles compared to extra-vacuolar compartments. The opposite effect of lower glucose contents, however, was described for overexpressors either of the endogenous or a homologous sugar beet transporter (BvIMP). Knock-out *erdl6* mutants, however, displayed increased seed yield with elevated sugar, protein, and lipid contents of mature seeds, as had been described for TMT1 overexpressors (Wingenter et al., 2010). It remains elusive if yield increase can be attributed to source or sink effects since *ERDL6* is expressed in leaves and seeds, according to the eFP browser (Winter et al., 2007). It also remains to be elucidated whether the mechanism of ERDL6-mediated transport is facilitation – as found for ESL1 – or is secondary transport.

Only very recently, the *Arabidopsis* vacuolar sugar exporter SWEET17 has been discovered with help of a quantitative genetics approach (“7” in **Figure 1;** Chardon et al., 2013). SWEET17 is the first protein among the SWEET transporter family localized to the tonoplast, all other SWEETs described previously were localized in the plasma membrane (Chen et al., 2010, 2012). Moreover, SWEET17 is the first fructose transporter, other SWEET family members facilitate diffusion of glucose or sucrose (Chen et al., 2010, 2012). The authors were able to demonstrate uptake or efflux of ^13^C-labeled fructose upon expression of SWEET17 in *Xenopus* oocytes. In *sweet17* mutants, fructose contents were enhanced compared to wild-type, and accumulation of fructose was more pronounced in nitrogen-limited or cold-stressed plants. Both growth conditions led to an elevated uptake of sugars into vacuoles mediated by TMTs (Wingenter et al., 2010; Schulz et al., 2011), and fructose appears to not be released from *sweet17* mutant vacuoles. SWEET17 expression was found in leaves, with the strongest level in xylem vascular tissue, pointing toward a function in fructose allocation within the plant. Accordingly, the only subtle phenotype of *sweet17* mutants was in the reduced size of flower stalks and slightly reduced seed yield (Chardon et al., 2013).

## PHLOEM LOADING, LEAKAGE, AND RETRIEVAL

When sucrose is exported from source leaves, it has to enter the phloem. Several strategies for the loading of sucrose (and other sugars and sugar alcohols) have been described (for a review, see Rennie and Turgeon, 2009). We have restricted ourselves here to apoplastic sucrose loading when describing the route sucrose takes from source leaves to sinks.

### SWEET-FACILITATED SUCROSE EFFLUX INTO THE APOPLAST

For many years it remained unknown how sucrose is released from mesophyll cells or phloem parenchyma cells into the apoplast prior to loading into the companion cell/sieve element complex. It was recently demonstrated that this release is facilitated by members of the SWEET transporter family, i.e., SWEET11 and -12 from *Arabidopsis* and SWEET11 and -14 from rice (“7” in **Figure 1;** Chen et al., 2012). Facilitation of sucrose efflux without energy consumption can occur due to the concentration gradient between the mesophyll cell/phloem parenchyma cell symplastic continuum and the apoplast has been described to be steep [40-, 150-, and 750-fold in spinach, barley (Lohaus et al., 1995), and sugar beet (Fondy and Geiger, 1977; Lohaus et al., 1994), respectively]. Closing of this gap in source sink carbon allocation was of great importance and accordingly was effusively acclaimed (Baker et al., 2012; Braun, 2012) not only because of the completion of a pathway but also due to enabling new perspectives with respect to genetic engineering of the SWEET proteins. Before sucrose transporting SWEETs had been discovered, other members of the family were found to facilitate efflux of glucose into the apoplast fulfilling a potential role in feeding pathogens residing in the cell wall (Chen et al., 2010) with maybe even higher potential for the agricultural industry (Sonnewald, 2011; Baker et al., 2012).

Whereas, single knock-out mutants of AtSWEET11 and -12 did not display any obvious altered phenotype, *Arabidopsis* *sweet11*/*sweet12* double mutants did display an expected phenotype of blocked phloem loading, i.e., smaller plants, elevated levels of leaf starch and sucrose, a reduced export of fixed ^14^C from leaves and reduced growth of sinks (e.g., roots; Chen et al., 2012). Rice *SWEET11* and -*14* were transcriptionally induced upon infection with *Xanthomonas oryzae* pv.* oryzae*. This induction is mediated through pathogen-borne effectors binding to *SWEET11* and -*14* promoters. Mutations in the binding sites led to resistance against the pathogen (Antony et al., 2010). This finding indicated that increasing SWEET activity would lead to more sucrose in the apoplast on which pathogens could feed and spread. Thus, an overexpression of SWEETs in non-infected plants would most likely also lead to an increase in apoplastic sucrose content. Whether or not SWEET overexpression could enhance source capacity would depend on the plasticity of SUC2/SUT1 transporter (“8” in **Figure 1**) activity importing sucrose from the apoplast into phloem companion cells in a sufficient capacity.

### PHLOEM LOADING OF SUCROSE

Sucrose is loaded into phloem companion cells and is transported in sieve elements along a hydrostatic pressure gradient driven by active sucrose loading. This enables a mass flow to sink organs with higher pressure in the source leaves and lower pressure in the sinks where sucrose is then unloaded from the phloem (Münch, 1930).

Because of the extreme difference in sucrose concentration between apoplast and phloem, sucrose loading against this gradient [630-, 700-, and 15,000-fold in spinach, barley (Lohaus et al., 1995), and sugar beet (Fondy and Geiger, 1977; Lohaus et al., 1994), respectively] must be energized. Apoplastic sucrose loading proceeds via a sucrose/H^+^-symport (Giaquinta, 1977a,b; Komor et al., 1977; Delrot and Bonnemain, 1981; Bush, 1989) using the proton gradient which is established by the plasma membrane-located P-type H^+^-ATPase. Direct evidence for an apoplastic step in sucrose loading into the phloem was provided by the expression of a yeast invertase in the apoplast (cell wall) of *Arabidopsis*, tobacco, tomato, and potato that led to increased carbohydrate levels in mature leaves and a concomitant decrease of photosynthesis (von Schaewen et al., 1990; Dickinson et al., 1991; Sonnewald et al., 1991; Heineke et al., 1992) resulting in reduced growth rates. These experiments demonstrated that only sucrose but not hexoses can be loaded into the phloem. The respective SUC2/SUT1 H^+^-cotransporters have been identified by functional complementation of yeast strains unable to grow on sucrose (Riesmeier et al., 1992, 1993b; Sauer and Stolz, 1994). Expression of these transporters was confined to the phloem (Riesmeier et al., 1993b; Gahrtz et al., 1994; Truernit and Sauer, 1995), and more precisely to companion cells (Stadler et al., 1995; Stadler and Sauer, 1996; Schmitt et al., 2008). Functional proof was established by antisense down-regulation of the SUT1 proteins from potato (Riesmeier et al., 1994; Kühn et al., 1996) and tobacco (Bürkle et al., 1998). Knock-out *suc2* *Arabidopsis* (Gottwald et al., 2000) and *sut1* maize (Slewinski et al., 2009, 2010) plants were characterized and displayed a similar phenotype to that found for the Solanaceae: carbohydrate accumulation in leaves, impaired growth and generally poor development of sinks. Over expression of a modified spinach SUT1 in potato did not lead to increased tuber starch content and yield (Leggewie et al., 2003). This could either be due to the use of a constitutive promoter to drive expression of the *SUT1* gene in mesophyll and phloem parenchyma cells that might have led to an unwanted re-uptake of sucrose from the apoplast into these cells and thus partial prevention of phloem loading. On the other hand, SUT1 activity might not be limiting for sucrose delivery to sink tissues or that potato tuber yield is not source-limited. *Arabidopsis* *suc2* mutants have been successfully complemented with the *Arabidopsis* *SUC1* gene expressed under control of the *AtSUC2* promoter. Some of these complemented lines displayed decreased leaf sucrose content indicative of more effective sucrose loading into the phloem (Wippel and Sauer, 2012). However, a possible impact on source capacity remains ambiguous since neither carbon export nor seed yield was comparatively analyzed in these plants. By over- and underexpressing the vacuolar TMT in *Arabidopsis* leaves, Wingenter et al. (2010) posed an indirect effect on *SUC2* transcripts, sucrose export from leaves and seed yield which have been found to positively correlate with TMT activity. However, it has to be elucidated whether or not TMT or other factors affect SUC2 activity which would in turn be accountable for altered source capacity.

### LEAKAGE FROM AND RETRIEVAL OF SUCROSE TRANSPORTED IN THE PHLOEM

Sucrose in the phloem is transported to the sink tissues from the leaves and partially leaks out of the phloem to nourish the surrounding tissue (Minchin et al., 1984; Minchin and Thorpe, 1987). Whereas the above-ground tissues are green and might be able to support themselves to a certain extent by photosynthesis, the below-ground tissues clearly depend on sucrose leakage out of the phloem. How and where sucrose leaks out of the phloem has not been analyzed in detail. The sieve element/companion cell complex seems to be symplastically isolated (Kempers et al., 1998) along the whole path of sucrose movement indicating that unloading from the sieve elements and/or companion cells into the apoplast has to be facilitated. It remains to be elucidated whether SWEETs (“7” in **Figure 1**) are expressed in sieve elements or companion cells along the phloem and are responsible for this function.

Having entered the apoplast, sucrose then has to be taken up into the symplast again to reach the sink cells that depend on sucrose import. On the other hand, a great proportion of the sucrose that leaked out is retrieved into the sieve element/companion cell complex (Hafke et al., 2005). In both directions, uptake must be energized (Ayre, 2011), and thus sucrose/H^+^-cotransporters are likely to fulfill this function. In *Arabidopsis*, the role of SUC2 (“8” in **Figure 1**) in retrieval of sucrose from the apoplast has been described by complementing the *suc2* mutant with a construct that restored SUC2 function in phloem loading (see above) but not in retrieval. The corresponding plants were smaller and exuded less ^14^C label from cut petioles when leaves photosynthesized in the presence of ^14^CO_2_. These findings are consistent with a role of SUC2 in retrieval of sucrose from the apoplast along the phloem (Srivastava et al., 2008). Analyses using ^11^C tracer studies support this role for AtSUC2 (Gould et al., 2012). In addition, localization in the sieve elements of *Arabidopsis* stems argues for a putative role of SUC3 in retrieval (“9” in **Figure 1;** Meyer et al., 2000, 2004). This is in addition to SUC2 which seems to retrieve sucrose back into companion cells. However, it remains unclear which sucrose/H^+^-cotransporters function in taking up sucrose to nourish the tissue surrounding the vasculature.

## CARBON UNLOADING AND STORAGE IN SINK ORGANS

Unloading of sucrose from the phloem can occur symplastically or apoplastically, depending not only on the plant species but also on tissue types and developmental stages.

### OIL STORAGE IN PLANTS

Most of the research on plants that store oil in their seed was performed on *Arabidopsis thaliana*. The important seed oil crop canola with cultivars of rapeseed (*Brassica napus*) or field mustard (*Brassica rapa*) belongs to the same family as *Arabidopsis*, the Brassicaceae. However, other plants with oil-storing seeds belong to the Asteraceae family, e.g., sunflower (*Helianthus annuus*), to the Fabaceae family, e.g., soybean (*Glycine max*), or the Arecaceae family, e.g., oil palms (*Elaeis guineensis*, *Elaeis oleifera*, *Attalea maripa*). Oil palms not only store oil in their seeds but also in their fruits.

However, sucrose delivered from source leaves via the phloem has to reach the terminal storage sinks, i.e., seeds and/or fruits, thereby crossing several membranes. Once there, sucrose undergoes a series of metabolic reactions to be stored as triacylglycerol (TAG) in the embryo again including several transport steps of intermediates across membranes. We will focus on *Arabidopsis* to describe the path of carbon from sucrose to TAG in the following paragraphs.

To define the symplastic domains, mobile and immobile versions of the GFP under the control of a variety of promoters as well as the low-molecular-weight fluorescent dye HPTS (8-hydroxypyrene-1,3,6-trisulfonate) were used (Schneidereit et al., 2003; Stadler et al., 2005a).

**FIGURE 2:**
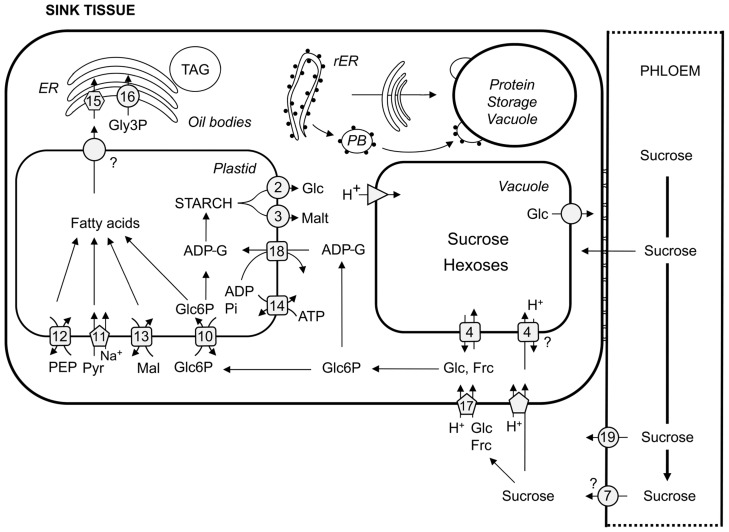
**Overview of carbon transport proteins in sink organs.** Transport steps in sieve elements and sink cells are depicted according to their respective mode of action. Squares represent antiporters, circles describe facilitators, pentagons depict symporters, hexagons represent ABC transporters, and triangles H^+^-ATPases/PPases. 2, pGlcT; 3, MEX; 4, TMT and VGT; 7, SWEETs; 10, GPT; 11, BASS2; 12, PPT, 13, DiT2; 14, NTT; 15, ABCA9; 16, Gly3P permease; 17, HT; 18, BT-1; 19, SUF1. (r)ER, (rough) endoplasmic reticulum; TAG, triacylglycerol; PB, protein body; PEP, phosphoenolpyruvate; Pyr, pyruvate; Mal, malate; Glc6P, glucose 6-phosphate; Pi, orthophosphate; ADP, adenosine diphosphate; ADP-G, ADP-glucose; Glc, glucose; Malt, maltose; Frc, fructose. Please refer to text for transporter abbreviations.

Having reached the funicular end of the phloem, sucrose is unloaded into an unloading domain near the funiculus, and post-phloem transport occurs symplastically into the seed coat (Imlau et al., 1999), or – more precisely – into the outer integument (Stadler et al., 2005b). Before anthesis, phloem unloading of sucrose switches from symplastic in ovule primordia cells to apoplastic in mature ovules and upon anthesis back to a symplastic mode (Werner et al., 2011). To progress into the inner integument, sucrose must reach the apoplast which is potentially facilitated by SWEETs. A sucrose/H^+^-symporter could also potentially transport sucrose into the inner integument. SUC3 has been discussed as a likely candidate (Stadler et al., 2005b) since it might be expressed in this cell layer (Meyer et al., 2004). However, the transfer of sucrose from the outer into the inner integument has not been analyzed in detail. The three layers of the inner integument form a symplastic continuum (Stadler et al., 2005b) isolated from the endosperm. After export from the inner integument, possibly mediated by a SWEET transporter, sucrose could be taken up into the endosperm by SUC5 which is expressed during seed development (Baud et al., 2005; Stadler et al., 2005b; Pommerrenig et al., 2013). Finally, before entering the embryo, sucrose has to be released into the apoplast separating endosperm and embryo. Again, one can speculate of a SWEET transporter family member (“7” in **Figure 2**) facilitating diffusion of sucrose out of the endosperm. Sucrose uptake into the embryo could be managed by three different SUC proteins (SUC3, -5, -9; **Figure 2**) that are expressed in different embryonic tissues at different developmental stages. With the help of a SUC9 promoter–GUS fusion, SUC9 expression could be detected in the mature embryo (Sivitz et al., 2007). More detailed analyses have been performed for the expression of both SUC5 and SUC3. In addition to the expression of SUC5 in the endosperm, torpedo and walking stick stage embryos express SUC5 in the epidermis of cotyledons (Pommerrenig et al., 2013). SUC3 is expressed in the suspensor which forms a symplastic continuum with the globular embryo. However, as early as the heart stage, the embryo proper is again symplastically isolated from the suspensor (Stadler et al., 2005b). From the torpedo stage on, SUC3 is expressed in the root epidermis which forms a symplastic connection with root and hypocotyls cells (Meyer et al., 2004; Stadler et al., 2005b). In almost fully developed embryos, SUC3 is still expressed in the root epidermis but seems to form a symplast with cells of the developing stele. Moreover, individual symplastically isolated cells of cotyledons express SUC3 (Stadler et al., 2005b). However, there seems to be redundancy in the described steps since none of the individual mutants displayed a phenotype different from wild-type. Whereas, there was no aberrant *suc3* phenotype detectable at all (Barth et al., 2003), *suc5* mutants displayed a transient decrease in seed TAG accumulation (Baud et al., 2005) which might be explained by the biotin transport function of SUC5 rather than the sucrose transport function (Pommerrenig et al., 2013). Under short-day conditions, *suc9* mutants show an early flowering phenotype but normally are indistinguishable from wild-type (Sivitz et al., 2007).

Once having reached the cytosol of embryo cells, sucrose is utilized to keep up metabolism and ultimately to build up TAG. TAG is formed from glycerol 3-phosphate (Gly3P) and acylated fatty acids in the endoplasmic reticulum (ER) in a series of reactions known as the Kennedy pathway (Kennedy, 1961). In plants, fatty acid synthesis takes place exclusively in plastids, and in heterotrophic tissues plastids depend on the import of carbon skeletons, energy and reduction power (Rawsthorne, 2002). Fatty acid synthesis starts from pyruvate that is first converted to acetyl-CoA by the plastidic pyruvate dehydrogenase complex. For synthesizing fatty acids from acetyl-CoA, ATP and NAD(P)H are required and thus have to be imported or generated within plastids. Carbon skeletons can be imported into plastids as glucose 6-phosphate (Glc6P), pyruvate, phosphoenolpyruvate (PEP), or malate. These metabolites are either intermediates of glycolysis, or in the case of malate, a tricarboxylic acid (TCA) cycle intermediate that can be exported from the mitochondria into the cytosol.

Glucose 6-phosphate is imported into plastids by the Glc6P/phosphate translocator (GPT; “10” in **Figure 2;** Kammerer et al., 1998) in counter-exchange with inorganic phosphate or triose phosphate generated by the oxidative pentose phosphate pathway (OPPP). Plastids isolated from young oilseed rape embryos utilize Glc6P better than pyruvate for fatty acid synthesis whereas plastids isolated from older embryos prefer pyruvate over Glc6P as a substrate for fatty acid synthesis (Eastmond and Rawsthorne, 2000). Moreover, imported Glc6P is also used for starch synthesis or as a substrate of the OPPP which is necessary for the production of NADPH required for fatty acid synthesis. Furthermore, partitioning of Glc6P into the different pathways is dependent on the developmental stage of the embryos (Eastmond and Rawsthorne, 2000). Seeds of *Arabidopsis* and oilseed rape transiently accumulate starch but completely degrade it until they reach maturity (da Silva et al., 1997; Baud et al., 2002; Andriotis et al., 2010a). Starch does not accumulate because of photosynthetic activity of the embryo and/or the seed coat, since seeds developing in darkened siliques also show transient starch accumulation (da Silva et al., 1997). It might be assumed that transient starch accumulation of seeds could be important for final TAG yield of embryos since *Arabidopsis* mutants defective in starch degradation (*sex1*; Yu et al., 2001) or biosynthesis (*phosphoglucomutase 1*, *pgm1*; Caspar et al., 1985) were found to contain 30 (Andriotis et al., 2010a) and 40% (Periappuram et al., 2000) less lipid content, respectively. In oilseed rape, the embryo-specific reduction of ADP-glucose pyrophosphorylase (AGPase) activity, i.e., down-regulation of starch synthesis, led to a delay in oil accumulation of developing seeds. However, mature seeds had wild-type seed oil content (Vigeolas et al., 2004). Andriotis et al. (2012) were able to show that starch turnover of the maternal plant is important for *Arabidopsis* seed TAG yield, and not starch turnover of the embryo itself, i.e., they found a reduction in (mainly nocturnal) source capacity causative for compromised seed TAG content. Nonetheless, Glc6P uptake and partitioning into fatty acid and the OPPP seems to be of major importance since restricting GPT activity by seed-specific antisense expression or RNA interference (RNAi) led to an arrest of embryo development despite the redundancy of carbon skeleton uptake systems of plastids. As mentioned above, pyruvate as a direct substrate for fatty acid synthesis can also be taken up by plastids. The plastid sodium-dependent pyruvate transporter BASS2 was found to import pyruvate into plastids (“11” in **Figure 2;** Furumoto et al., 2011). Alternatively, pyruvate can be produced from PEP that could either be formed within the plastid stroma (Flügge et al., 2011) or imported by the PEP/phosphate translocator (PPT; “12” in **Figure 2;** Fischer et al., 1997). Moreover, malate can also serve as a precursor for stromal pyruvate. NADP malic enzyme produces pyruvate, CO_2_ and reduction power from malate that could be imported into plastids by the dicarboxylate transporter DiT2 (“13” in **Figure 2;** Renné et al., 2003). However, none of these substrates can fuel the OPPP. Therefore, compromised generation of sufficient reduction power might explain the severity of the GPT antisense or RNAi plant phenotype (Andriotis et al., 2010b). Whereas, in *bass2* mutants there was no aberrant phenotype under normal growth conditions, though these were not analyzed for fatty acid or seed TAG content (Furumoto et al., 2011), *ppt1* (*cue1*; Li et al., 1995) mutants display a reticulate phenotype that spoils meaningful yield analyses. The supposed reason for the apparent phenotype is that PPT1 is not only involved in substrate supply for fatty acid synthesis but also important in secondary metabolite production (Streatfield et al., 1999; Voll et al., 2003).

DiT2 is essential for photorespiration, in that, *dit2* (*dct*; Somerville and Ogren, 1983; Somerville and Somerville, 1985) mutants do not re-assimilate ammonium released during photorespiration due to a lack of glutamate export from plastids and do not survive when grown under ambient CO_2_ concentrations.

As mentioned above, energy has to be imported into heterotrophic plastids to drive – amongst other processes – transient starch synthesis and optimal fatty acid synthesis. Plastids import ATP via adenylate translocators (NTTs; “14” in **Figure 2;** Kampfenkel et al., 1995) in counter-exchange with ADP and orthophosphate (Trentmann et al., 2008). *Arabidopsis* contains two NTTs and indeed *ntt1/ntt2* double mutants are impaired in seed protein and lipid contents and in seed yield (Reiser et al., 2004).

Fatty acids up to a length of 18 carbon atoms are produced in plastids. Incorporation of acylated fatty acids into TAG occurs in the ER (Li-Beisson et al., 2013). Hence, fatty acids have to be exported from plastids presumably by the recently identified fatty acid exporter FAX1 (K. Philippar, personal communication), and imported into the ER, and prior to incorporation into TAG have to be acylated by LACS proteins at the plastid outer envelope membrane (Shockey et al., 2002). To prevent inhibition of plastid metabolite transporters and thus fatty acid synthesis by cytosolic acyl-CoAs (Fox et al., 2001) and to assure efficient TAG production from acyl-CoAs, uptake into the ER should be rapid. Often reported spatial proximity of plastids or plastid stromules (filamentous tubules increasing the plastid surface area) and the ER (Schattat et al., 2011) might support a short retention time of acyl-CoA molecules in the cytosol. Recently, a transporter taking up free and acylated fatty acids into the ER has been described from *Arabidopsis*. The ABC transporter ABCA9 (“15” in **Figure 2**) has been found to localize to the ER, *abca9* mutants had decreased seed weight, total lipid and TAG and were defective in effectively taking up ^14^C-labeled oleoyl-CoA and oleic acid. Moreover, constitutive overexpression of *ABCA9* driven by a constitutive promoter led to enlarged seeds with increased dry weight. The TAG content per seed was increased by 40% compared to wild-type. Since silique number per plant and seed number per silique were similar, overexpression of ABCA9 increased total seed oil per plant (Kim et al., 2013). This finding strongly argues for *Arabidopsis* yield to be sink-limited as it seems unlikely that the present expression of ABCA9 in leaves has a positive effect on source capacity. Moreover, it demonstrates that TAG production in the ER of embryo cells is limited by the supply with fatty acids rather than the Gly3P moiety of TAG. Nonetheless, Gly3P has to be imported into or present in the ER for TAG synthesis. This might be achieved by a member of the Gly3P permease family (“16” in **Figure 2;** Ramaiah et al., 2011), by phosphorylation of glycerol and/or by reduction of dihydroxyacetone phosphate. After having passed through the Kennedy pathway, synthesized TAG is stored in oil bodies (Hsieh and Huang, 2004; He and Wu, 2009) and can be used to fuel seedling establishment upon germination.

### SUGAR STORAGE IN PLANTS

Some crop plants store sugars in tap roots, stems, and fruits, among them are sugar beet (*Beta vulgaris*), a member of the Amaranthaceae family, sugarcane (*Saccharum* hybrids) belonging to the Poaceae, grape (*Vitis vinifera*), a member of the Vitaceae family, and tomato (*Solanum lycopersicum*) belonging to the Solanaceae family. The former two species store sucrose whereas the latter two mainly store hexoses in vacuoles of roots, storage parenchyma, and fruit cells. Sucrose unloaded from the phloem has to reach these cells, and upon presence of an apoplastic step has to be imported into the cells either as sucrose or, when cleaved by an apoplastic invertase, as hexoses. Moreover, tonoplast transporters have to import sugars into the vacuole.

In young sugar beet tap roots growing with a rapid relative growth rate, sucrose unloaded from the phloem is cleaved by extracellular invertases indicating an apoplastic step in post-phloem transport. In older tap roots, invertase activities decrease and sucrose synthase activity increases (Klotz and Finger, 2002; Godt and Roitsch, 2006). Moreover, in experiments with ^14^C-labeled sucrose fed to mature tap root tissue, there was little evidence for a hydrolytic step preceding sucrose uptake. Furthermore, the uptake of sucrose occurs against a concentration gradient, and thus requires metabolic energy (Giaquinta, 1979; Wyse, 1979). Vacuolar ATPase inhibitors prevented uptake of sucrose whereas plasma membrane ATPase inhibitors did not (Saftner et al., 1983) indicating that post-phloem sucrose transport into storage cells of mature tap roots occurs symplastically whereas import into the vacuole is energized by a V-type H^+^-ATPase. This view is supported by analyses of tonoplast vesicles prepared from red beet root vacuoles which were found to hydrolyze ATP during sucrose transport (Getz, 1991). Sucrose-induced proton export occurred in a 1:1 stoichiometry (Getz and Klein, 1995) consistent with the idea of a sucrose/H^+^-antiporter mediating the import of sucrose into storage vacuoles of sugar beet tap roots. Chiou and Bush (1996) described a tonoplast-localized transport protein expressed in leaves and tap roots. However, this transporter turned out to be a homolog of ERDL6, a vacuolar glucose exporter described above (**Figure 2;** Poschet et al., 2011).

In sugarcane, repeated sucrose breakdown and re-synthesis before storage in stem parenchyma cells has been described (Whittaker and Botha, 1997; Zhu et al., 1997). This “futile cycling” decreases with tissue maturity (Bindon and Botha, 2002; Uys et al., 2007). In mature internodes of stems sucrose seems to be symplastically unloaded since stem parenchyma cells are separated from the phloem by lignified and suberized cells preventing apoplastic unloading. Movement of the fluorescent tracer dye carboxyfluorescein from phloem to stem parenchyma cells indeed indicates symplastic connections. In younger internodes, a SUC2 ortholog is expressed in cells that are destined to be lignified (Rae et al., 2005) indicating an apoplastic step of sucrose unloading at this developmental stage. In mature internodes, sucrose was modeled to accumulate in vacuoles against a concentration gradient (Uys et al., 2007). This view was further substantiated by the detection of specialized acidic vacuoles with V-type H^+^-ATPases in the stem (Rae et al., 2009), presumably maintaining the proton gradient that might be necessary to accumulate sucrose (against a concentration gradient) in a suggested proton antiport manner (“4” in **Figure 2;** Grof and Campbell, 2001). Both sugar beet and sugarcane have not been functionally analyzed regarding yield limitation, probably partially due to their resistance to be efficiently transformed.

Grape berries also undergo a developmental switch in unloading and post-phloem transport of sucrose. In contrast to sucrose-storing sugar beet and sugarcane, they shift from symplastic to apoplastic sucrose unloading upon onset of ripening as revealed by fluorescent dye analyses. Cell wall invertase activity increases at the onset of ripening, further substantiating apoplastic unloading at this developmental stage (Zhang et al., 2006). Moreover, Fillion et al. (1999) found that the STP-type hexose transporter VvHT1 (“17” in **Figure 2**) is expressed during ripening indicating that some of the sucrose might be apoplastically cleaved by cell wall invertases before being taken up into the cytosol. Sugars accumulate mainly as hexoses in vacuoles of berry cells upon onset of ripening (Davies and Robinson, 1996). In a comprehensive approach, Afoufa-Bastien et al. (2010) gave a phylogenetic overview on sugar transporters and also analyzed their expression. Upon the onset of ripening, several hexose transporters, TMTs and SUC11/12 were expressed in berries. SUC11/12 expression in ripening berries was also found in an independent study (Manning et al., 2001), and VvTMT2 (“4” in **Figure 2**) expression has also been identified as ripening-related by Cakir and Giachino (2012). Taken together, expression analyses of several transporters support the finding that vacuolar storage of hexoses occurs by a switch to apoplastic unloading of sucrose upon onset of berry ripening. However, whether source- or sink capacity determines yield of grape berries remains elusive.

Tomato fruits and grape berries behave similarly with respect to sucrose unloading and post-phloem transport. In young tomato fruits, when starch content is built up in plastids of the columella and inner pericarp region of the fruit (Robinson et al., 1988; Schaffer and Petreikov, 1997), unloading is mainly symplastic. Upon the developmental shift to rapid hexose accumulation, also in the outer pericarp region, an apoplastic step is likely to occur (Ruan and Patrick, 1995) and is linked to an energized uptake of hexoses (“17” in **Figure 2;** Damon et al., 1988; Ruan et al., 1997) and sucrose (**Figure 2;** Damon et al., 1988). Similar to sugarcane, “futile cycles” involving invertase, sucrose synthase and starch synthesis and degradation seem to be abundant in tomato fruits to sustain sink capacity (Nguyen-Quoc and Foyer, 2001). The importance of transient starch accumulation for yield – unlike the above mentioned Brassicaceae seeds – was demonstrated by Baxter et al. (2005) who analyzed an introgression line with a fruit apoplastic invertase from the wild species *Solanum pennellii* introgressed into *Solanum lycopersicum*. Here, a dramatic increase in starch accumulation in early developmental stages was observed pointing toward partial apoplastic unloading already at younger developmental stages. Introgression lines had higher total soluble solids (TSS), a major determinant of fruit quality for processing (Baxter et al., 2005). More direct evidence for the importance of transient starch accumulation has been obtained by Petreikov et al. (2009) who developed and characterized introgression lines of *Solanum lycopersicum* harboring a wild species *Solanum habrochaites* allele for the regulatory large subunit of AGPase. This led to a large transient increase in starch which accounted for the enhanced amount of TSS and also an increase in fruit size. However, when hexose transporter- (STP-type) mediated uptake of hexoses from the apoplast was impaired by antisense expression of tomato *HT1*, *HT2*, and *HT3* (“17” in **Figure 2;** McCurdy et al., 2010), yield was decreased. Taken together, these findings point toward tomato yield being limited by sink capacity that can be increased when limitations of photoassimilate import into fruits are abolished.

### STARCH STORAGE IN PLANTS

Apart from soybean and sugarcane, the most important crops store starch, among them the grasses corn (*Zea mays*), rice (*Oryza sativa*), and wheat (*Triticum aestivum*) belonging to the Poaceae family, and potato (*Solanum tuberosum*), a member of the Solanaceae family. Other starch-storing staple foods are yams (*Dioscorea* sp.), belonging to the Dioscoreaceae, or cassava (*Manihot esculenta*), a member of the Euphorbiaceae. Grasses store starch in the endosperm of their caryopses, popularly called the “grain.” Potato, yams, and cassava store starch in tubers, the former tuber is derived from the sprout, the two latter tubers from roots.

In grasses, sucrose released from the phloem in grains symplastically reaches the maternal side of the maternal/filial interface, in wheat described as nucellar projection (Fisher and Cash-Clark, 2000; Patrick and Offler, 2001; Krishnan and Dayanandan, 2003). At the interface which appears as an endosperm cavity in wheat grains, cells can specialize on the maternal, as well as, on the filial side to transfer cells (characterized by cell wall in growths) to maximize plasma membrane size for release and uptake of sugars. In corn missing such an endosperm cavity, the filial cells can undergo specialization to transfer cells (Patrick and Offler, 2001; Gómez et al., 2002; Krishnan and Dayanandan, 2003). It can be hypothesized that sugar unloading is mediated by SWEETs (“7” in **Figure 2**). To enter the filial tissue, sugars have to be taken up from the apoplast, likely to be mediated by sucrose- or hexose/H^+^-symporters (**Figure 2**). In the endosperm, symplastic connections between uptake and storage cells exist (Patrick and Offler, 2001).

In cereal endosperm, starch synthesis differs from other starch-storing organs in that most of the AGPase activity is extraplastidic, i.e., cytosolic (Denyer et al., 1996; Thorbjörnsen et al., 1996; Beckles et al., 2001; James et al., 2003), requiring an additional uptake system for carbon skeletons into plastids supplementary to the GPT (“10” in **Figure 2;** Kammerer et al., 1998). Externally supplied ADP-glucose (ADP-G) was found to drive starch synthesis in isolated maize endosperm amyloplasts (Möhlmann et al., 1997) while Shannon et al. (1998) found Brittle-1 (BT-1; “18” in **Figure 2;** Sullivan and Kaneko, 1995) to be responsible for the uptake of ADP-G into maize endosperm amyloplasts by including the *bt-1* mutant in the analyses. Direct uptake measurements with the BT-1 from maize endosperm heterologously expressed in *Escherichia coli* cells revealed ADP-G uptake in counter-exchange with ADP (Kirchberger et al., 2007). Several approaches to increase starch yield of wheat and corn grains were successful when cytosolic AGPase activity was increased (Smidansky et al., 2002; Wang et al., 2007; Li et al., 2011), indicating that ADP-G import and thus BT-1 activity might not be limiting for starch yield. However, in contrast to the notion that photosynthesis is the limiting factor for increased yield (e.g., Long et al., 2006; Makino, 2011), these results suggest that sink- rather than source capacity limits grain starch yield in cereals.

In potato, starch is stored in tubers from which the plant can vegetatively, i.e., clonally propagate without a filial generation. Tubers are formed from stolons, below-ground lateral shoots. Upon induction of tuberization (Rodrïguez-Falcón et al., 2006), stolons start to swell at their apical hook. In non-tuberized stolons, sucrose transported in the phloem is unloaded apoplastically. This view is supported by experiments using carboxyfluorescein trapped in the phloem and ^14^C-labeled assimilates, mainly sucrose, which were evenly distributed in the stolon, i.e., also outside the phloem. Upon tuberization, carboxyfluorescein was able to leave the phloem, but only in the swollen part of the stolon, indicative for symplastic unloading. Taken together, tuberization involves a switch from apoplastic to symplastic phloem unloading (Viola et al., 2001). However, this switch is likely to be incomplete because otherwise an impact of apoplastically localized yeast invertase expressed under control of a tuber-specific promoter on metabolism could not be explained (Sonnewald et al., 1997; Hajirezaei et al., 2000). Alternatively, symplastically unloaded sucrose might leak out of sink cells. Again, a yet to be identified member of the SWEET family transporter (“7” in **Figure 2**) may be discussed as the facilitator of apoplastic phloem unloading or as the transporter mediating leakage of sucrose. Whether mainly sucrose or hexoses – produced from sucrose by cell wall invertases – are imported into sink cells probably by sucrose- or hexose/H^+^-symporters (**Figure 2**) remains to be elucidated. It is also unknown which transporters are engaged in this process. Sucrose entering sink cells via the apoplast or plasmodesmata might be further metabolized by sucrose synthase, since invertase activity is gradually reduced, while sucrose synthase activity yielding UPG-glucose and fructose increased during development (Hajirezaei et al., 2000). A part of the imported sucrose is further used to drive energy production indispensable for anabolic processes such as starch synthesis in heterotrophic tuber tissue. To synthesize starch, carbon skeletons and energy are imported into amyloplasts. Glc6P is imported by a GPT (“10” in **Figure 2**) in counter-exchange with orthophosphate or triose phosphate, and ATP generated in mitochondria is imported by the adenylate translocator NTT (“14” in **Figure 2**) in counter-exchange with ADP and orthophosphate. Shortage of energy import into amyloplasts as revealed by antisense repression of the adenylate translocator, led to compromised tuber starch content and yield (Tjaden et al., 1998). Overexpression, however, had no impact on starch yield, irrespective of the promoter, constitutive (Tjaden et al., 1998) or tuber-specific (Zhang et al., 2008), that drove expression of the transgene. Tuber-specific overexpression of a pea GPT also had no impact on tuber starch yield. Only when both carbon and energy supply to plastids was increased simultaneously, mediated by overexpression of GPT and NTT, tuber starch content and yield increased (Zhang et al., 2008). This indicated that import of carbon and energy co-limit starch yield which appears sink- rather than source-limited under these growth conditions. Moreover, a combined enhancement of sink- and source capacities led to even higher starch yield of triple-transgenic potato plants. The overexpression of GPT and NTT in tubers and additionally either overexpression of an *Escherichia coli* pyrophosphatase in mesophyll cytosol or reduced AGPase activity in leaves increased sucrose synthesis in source tissues (Jonik et al., 2012). Without simultaneously increasing sink capacity, neither leaf-specific antisense repression of AGPase (Leidreiter et al., 1995) nor mesophyll-specific cytosolic overexpression of pyrophosphatase (Jonik et al., 2012) – the latter analyzed in field trials and in the greenhouse – led to an increase in tuber starch yield indicating once more that potato tuber starch yield is sink-limited.

### PROTEIN STORAGE IN PLANTS

Members of the Fabaceae family store protein in their seeds, which represent the main plant source for human nitrogen nutrition. However, soybean (*G. max*) is primarily grown to provide oil from seeds, and pea (*Pisum sativum*) seeds contain more starch than protein. Other crop members of the family are bean (*Phaseolus vulgaris*), chickpea (*Cicer arietinum*), and peanut (*Arachis hypogaea*). There are species of families other than Fabaceae which contain reasonable amounts of storage protein in their harvested organs. However, these are mainly grown for their starch (e.g., potato, corn) or oil (e.g., canola). In order to store protein ample nitrogen must be available. Consistently, members of the Fabaceae family living in symbiosis with rhizobial bacteria store protein in seeds as the symbiotic bacteria fix atmospheric molecular nitrogen and supply the host plant with sufficient reduced nitrogen.

In legumes, protein is stored in the embryo in structures known as protein storage vacuoles. Storage proteins are translated at the ribosomes of the rough ER and are co-translationally imported into the ER. The main route from the ER to protein storage vacuoles is via the Golgi (Herman and Larkins, 1999). However, the alternative pathway, protein bodies budding off the ER and either remaining in the cytosol or fusing with the protein storage vacuole (as previously described in cereals) also seems to exist in legumes (**Figure 2;** Herman and Larkins, 1999; Vitale and Ceriotti, 2004; Abirached-Darmency et al., 2012).

Sucrose (and amino acid) unloading from the phloem in legume seeds occurs symplastically. Analyses with fluorescent dyes indicate that sucrose is symplastically transported to the maternal release site, the ground- or thin-walled parenchyma (Tegeder et al., 1999; van Dongen et al., 2003). Sucrose is either simply or through specialized transfer cells released into the apoplast dependent on the legume species (Patrick and Offler, 2001; van Dongen et al., 2003). At the younger stages, legume seeds contain a liquid endosperm which acts as a buffer for sucrose and glutamine for the developing embryo (Melkus et al., 2009). At the expense of embryo growth, the endosperm is substantially degraded during development (Patrick and Offler, 2001). At later developmental stages, sucrose is released from maternal tissue to the apoplast and is taken up by embryo epidermal transfer cells (Weber et al., 1997; Tegeder et al., 1999). With the discovery of a new class of sucrose facilitators from pea and bean, called SUFs, expressed at the maternal release site, sucrose release into the apoplast is probably mediated by these transporters (“19” in **Figure 2;** Zhou et al., 2007). In these same cells, the sucrose/H^+^-symporter SUT1 was found to be expressed and believed to likely function in seed coat sucrose retrieval (Tegeder et al., 1999; Zhou et al., 2007). At the filial side, uptake of sucrose by the embryo epidermis is mediated by SUT1 (**Figure 2**), and uptake is energized by P-type H^+^-ATPases which are both co-expressed in embryo transfer cells (Tegeder et al., 1999). The embryo communicates the demand for sucrose uptake from the apoplast by the internal sugar level. Sucrose uptake fluxes have been found to be negatively correlated with pool sizes of intracellular sugars, while *SUT1* transcripts were sensitive to sugar levels (i.e., when the embryo contains sufficient sugars, *SUT1* is transcriptionally down-regulated), which in turn led to decreased sucrose uptake (Zhou et al., 2009). To increase the uptake of sucrose into pea embryos, potato SUT1 was overexpressed using a storage parenchyma-specific promoter which resulted in increased sucrose uptake and accelerated growth rates of the embryo. However, final seed weight of transgenic plants was similar to the wild-type. The authors speculate that overexpression at the primary site of sucrose uptake, the epidermal transfer cells, might have led to more significant results (Rosche et al., 2002). In a different transgenic approach, seed protein content in mature seeds was increased. Rolletschek et al. (2007) antisense inhibited the *Vicia narbonensis* *GPT1* (“10” in **Figure 2**) using a seed-specific promoter. As expected, flux of carbon into plastids and thus into starch and (mainly structural) lipids was found to be decreased. A simultaneous increase in seed protein, however, was not predictable. The authors explain this finding with increased expression of the amino acid permease *AAP1* in transgenic embryos potentially due to lower glutamine concentrations during the main protein storage phase. Glutamine was previously described to repress *AAP1* transcriptionally (Miranda et al., 2001). Indeed, when AAP1 was overexpressed in seeds of *Vicia narbonensis* and pea, sink strength for amino acids was increased and the amounts of total nitrogen and protein (mainly storage globulins) were increased (Rolletschek et al., 2005).

## CONCLUDING REMARKS

This review on plant carbon allocation and transport unequivocally demonstrates the increasing importance of sub- and intercellular, as well as, long-distance transport processes. Given the impact of several of the described modifications on yield of various crop plants, we suggest continued work on several of the insinuated and not yet conceived transport steps within plants which frequently turn out to be the rate-limiting steps for the production of valuable compounds in storage sinks.

## Conflict of Interest Statement

The authors declare that the research was conducted in the absence of any commercial or financial relationships that could be construed as a potential conflict of interest.

## References

[B1] Abirached-Darmency M. Dessaint F. Benlicha E. Schneider C. (2012). Biogenesis of protein bodies during vicilin accumulation in *Medicago truncatula* immature seeds. *BMC Res. Notes * 5: 409 10.1186/1756-0500-5-409 PMC3431269 22862819

[B2] Afoufa-Bastien D. Medici A. Jeauffre J. Coutos-Thévenot P. Lemoine R. Atanassova R. (2010) The *Vitis vinifera* sugar transporter gene family: phylogenetic overview and macroarray expression profiling. *BMC Plant Biol. * 10: 245 10.1186/1471-2229-10-245 PMC3095327 21073695

[B3] Ainsworth E. A. Bush D. R. (2011) Carbohydrate export from the leaf: a highly regulated process and target to enhance photosynthesis and productivity. *Plant Physiol.* 155 64–69 10.1104/pp.110.167684 20971857 PMC3075787

[B4] Aluri S Büttner M. (2007) Identification and functional expression of the *Arabidopsis* *thaliana *vacuolar glucose transporter 1 and its role in seed germination and flowering. *Proc. Natl. Acad. Sci. U.S.A.* 104 2537–2542 10.1073/pnas.0610278104 17284600 PMC1892959

[B5] Andriotis V. M. E. Pike M. J. Kular B. Rawsthorne S. Smith A. M. (2010a) Starch turnover in developing oilseed embryos. *New Phytol.* 187 791–804 10.1111/j.1469-8137.2010.03311.x 20546137

[B6] Andriotis V. M. E. Pike M. J. Bunnewell S. Hills M. J. Smith A. M. (2010b) The plastidial glucose-6-phosphate/phosphate antiporter GPT1 is essential for morphogenesis in *Arabidopsis* embryos. *Plant J.* 64 128–139 10.1111/j.1365-313X.2010.04313.x 20659277

[B7] Andriotis V. M. E. Pike M. J. Schwarz S. L. Rawsthorne S. Wang T. L. Smith A. M. (2012) Altered starch turnover in the maternal plant has major effects on *Arabidopsis* fruit growth and seed composition. *Plant Physiol.* 160 1175–1186 10.1104/pp.112.205062 22942388 PMC3490605

[B8] Antony G. Zhou J. Huang S. Li T. Liu B. White F. (2010) Rice xa13 recessive resistance to bacterial blight is defeated by induction of the disease susceptibility gene Os-11N3. *Plant Cell* 22 3864–3876 10.1105/tpc.110.078964 21098734 PMC3015117

[B9] Ayre B. G. (2011) Membrane-transport systems for sucrose in relation to whole-plant carbon partitioning. *Mol. Plant* 4 377–394 10.1093/mp/ssr014 21502663

[B10] Baker R. F. Leacvh K. A. Braun D. M. (2012) SWEET as sugar: new sucrose effluxers in plants. *Mol. Plant* 5 766–768 10.1093/mp/SSS054 22815540

[B11] Barth I. Meyer S. Sauer N. (2003) PmSUC3: characterization of a SUT2/SUC3-type sucrose transporter from *Plantago major*. *Plant Cell* 15 1375–1385 10.1105/tpc.010967 12782730 PMC156373

[B12] Baud S. Boutin J.-P. Miquel M. Lepiniec L. Rochat C. (2002) An integrated overview of seed development in *Arabidopsis thaliana* ecotype WS. *Plant Physiol. Biochem.* 40 151–160 10.1016/S0981-9428(01)01350-X

[B13] Baud S. Wuillème S. Lemoine R. Kronenberger J. Caboche M. Lepiniec L. (2005) The AtSUC5 sucrose transporter specifically expressed in the endosperm is involved in early seed development in *Arabidopsis*. *Plant J.* 43 824–836 16146522 10.1111/j.1365-313X.2005.02496.x

[B14] Baxter C. J. Carrari F. Bauke A. Overy S. Hill S. A. Quick P. W. (2005) Fruit carbohydrate metabolism in an introgressed line of tomato with increased fruit soluble solids. *Plant Cell Physiol.* 46 425–437 10.1093/pcp/pci040 15695458

[B15] Beckles D. M. Smith A. M ap Rees T. (2001) A cytosolic ADP-glucose pyrophosphorylase is a feature of graminaceous endosperms, but not of other starch-storing organs. *Plant Physiol.* 125 818–827 10.1104/pp.125.2.818 11161039 PMC64883

[B16] Bindon K. A. Botha F. C. (2002) Carbon allocation to the insoluble fraction, respiration and triose-phosphate cycling in the sugarcane culm. *Physiol. Plant.* 116 12–19 10.1034/j.1399-3054.2002.1160102.x 12207657

[B17] Braun D. M. (2012) SWEET! The pathway is complete. *Science* 335 173–174 10.1126/science.1216828 22246760

[B18] Bürkle L. Hibberd J. Quick W. P. Kühn C. Hirner B. Frommer W. B. (1998) The H+-sucrose cotransporter NtSUT1 is essential for sugar export from tobacco leaves. *Plant Physiol.* 118 59–68 9733526 10.1104/pp.118.1.59 PMC34874

[B19] Bush D. R. (1989) Proton-coupled sucrose transport in plasmalemma vesicles isolated from sugar beet (*Beta vulgaris* L. cv Great Western) leaves.* Plant Physiol.* 89 1318–1323 10.1104/pp.89.4.1318 PMC1056015 16666703

[B20] Büttner M. (2007) The monosaccharide transporter(-like) gene family in *Arabidopsis*. *FEBS Lett.* 581 2318–2324 17379213 10.1016/j.febslet.2007.03.016

[B21] Büttner M. (2010) The *Arabidopsis* sugar transporter (AtSTP) family: an update. *Plant Biol. Suppl.* 1 35–41 10.1111/j.1438-8677.2010.00383.x 20712619

[B22] Cakir B Giachino R. R. A. (2012) VvTMT2 encodes a putative tonoplast monosaccharide transporter expressed during grape berry (*Vitis vinifera* cv. Sultanine)**ripening*. Plant Omics J.* 5 576–583

[B23] Caspar T. Huber S. C. Somerville C. (1985) Alterations in growth, photosynthesis, and respiration in a starchless mutant of *Arabidopsis thaliana* (L.) deficient in chloroplast phosphoglucomutase activity. *Plant Physiol.* 79 11–17 10.1104/pp.79.1.11 16664354 PMC1074821

[B24] Chardon F. Bedu M. Calenge F. Klemens P. A. W. Spinner L. Clement G. (2013) Leaf fructose content is controlled by the vacuolar transporter SWEET17 in *Arabidopsis*. *Curr. Biol*. 23 697–702 10.1016/j.cub.2013.03.021 23583552

[B25] Chen L.-Q. Hou B.-H. Lalonde S. Takanaga H. Hartung M. L. Qu X.-Q. (2010) Sugar transporters for intercellular exchange and nutrition of pathogens. *Nature* 468 527–534 10.1038/nature09606 21107422 PMC3000469

[B26] Chen L.-Q. Qu X.-Q. Hou B.-H. Sosso D. Osorio S. Fernie A. R. (2012) Sucrose efflux mediated by SWEET proteins as a key step for phloem transport. *Science* 335 207–211 10.1126/science.1213351 22157085

[B27] Chincinska I. A. Liesche J. Krügel U. Michalska J. Geigenberger P. Grimm B. (2008) Sucrose transporter StSUT4 from potato affects flowering, tuberization, and shade avoidance response. *Plant Physiol.* 146 515–528 18083796 10.1104/pp.107.112334 PMC2245842

[B28] Chiou T. J. Bush D. R. (1996) Molecular cloning, immunochemical localization to the vacuole, and expression in transgenic yeast and tobacco of a putative sugar transporter from sugar beet. *Plant Physiol.* 110 511–520 10.1104/pp.110.2.511 8742332 PMC157746

[B29] Cho M.-H. Jang A. Bhoo S. H. Jeon J.-S. Hahn T.-R. (2012) Manipulation of triose phosphate/phosphate translocator and cytosolic fructose-1,6-bisphosphatase, the key components in photosynthetic sucrose synthesis, enhance the source capacity of transgenic *Arabidopsis* plants. *Photosynth. Res.* 111 261–268 10.1007/s11120-012-9720-2 22297909

[B30] Cho M.-H. Lim H. Shin D. H. Jeon J.-S. Bhoo S. H. Park Y.-I. (2011) Role of plastidic glucose transporters in the export of starch degradation products from the chloroplasts in *Arabidopsis thaliana*. *New Phytol.* 190 101–112 10.1111/j.1469-8137.2010.03580.x 21175634

[B31] Damon S. Hewitt J. Nieder M. Bennett A. B. (1988) Sink metabolism in tomato fruit. II. Phloem unloading and sugar uptake*. Plant Physiol.* 87 731–736 10.1104/pp.87.3.731 PMC1054829 16666216

[B32] da Silva P. M. F.R. Eastmond P. J. Hill L. M. Smith A. M. Rawsthorne S. (1997) Starch metabolism in developing embryos of oilseed rape. *Planta* 203 480–487 10.1007/s004250050217

[B33] Davies C. Robinson S. P. (1996) Sugar accumulation in grape berries. Cloning of two putative vacuolar invertase cDNAs and their expression in grapevine tissues.*Plant Physiol*. 111 275–283 10.1104/pp.111.1.275 PMC157835 8685267

[B34] Delrot S. Bonnemain J.-L. (1981) Involvement of protons as a substrate for the sucrose carrier during phloem loading in *Vicia faba* leaves. *Plant Physiol.* 67 560–564 10.1104/pp.67.3.560 16661714 PMC425725

[B35] Denyer K. Dunlap F. Thorbjörnsen T. Keeling P. Smith A. M. (1996) The major form of ADP-glucose pyrophosphorylase in maize endosperm is extra-plastidial. *Plant Physiol.* 112 779–785 8883389 10.1104/pp.112.2.779 PMC158002

[B36] Dickinson C. D. Altabella T. Chrispeels M. J. (1991) Slow-growth phenotype of transgenic tomato expressing apoplastic invertase. *Plant Physiol.* 95 420–425 10.1104/pp.95.2.420 16668000 PMC1077547

[B37] Eastmond P. J. Rawsthorne S. (2000) Coordinate changes in carbon partitioning and plastidial metabolism during the development of oilseed rape embryos. *Plant Physiol.* 122 767–774 10.1104/pp.122.3.767 10712540 PMC58912

[B38] Endler A. Meyer S. Schelbert S. Schneider T. Weschke W. Peters S. W. (2006) Identification of a vacuolar sucrose transporter in barley and *Arabidopsis* mesophyll cells by a tonoplast proteomic approach. *Plant Physiol.* 141 196–207 10.1104/pp.106.079533 16581873 PMC1459324

[B39] Eom J.-S. Cho J.-I. Reinders A. Lee S.-W. Yoo Y. Tuan P. Q. (2011) Impaired function of the tonoplast-localized sucrose transporter in rice, OsSUT2, limits the transport of vacuolar reserve sucrose and affects plant growth. *Plant Physiol.* 157 109–119 10.1104/pp.111.176982 21771914 PMC3165862

[B40] Fillion L. Ageorges A. Picaud S. Coutos-Thévenot P. Lemoine R. Romieu C. (1999) Cloning and expression of a hexose transporter gene expressed during ripening of grape berry. *Plant Physiol.* 120 1083–1093 10444092 10.1104/pp.120.4.1083 PMC59342

[B41] Fischer K. Kammerer B. Gutensohn M. Arbinger B. Weber A. Häusler R. E. (1997) A new class of plastidic phosphate translocators: a putative link between primary and secondary metabolism by the phosphoenolpyruvate/phosphate antiporter. *Plant Cell* 9 453–462 9090886 10.1105/tpc.9.3.453 PMC156930

[B42] Fisher D. B. Cash-Clark C. E. (2000) Sieve tube unloading and post-phloem transport of fluorescent tracers and proteins injected into sieve tubes via severed aphid stylets. *Plant Physiol.* 123 125–137 10.1104/pp.123.1.125 10806231 PMC58988

[B43] Fliege R. Flügge U.-I. Werdan K. Heldt H. W. (1978) Specific transport of inorganic phosphate, 3-phosphoglycerate and triosephosphates across the inner membrane of the envelope in spinach chloroplasts. *Biochim. Biophys. Acta* 502 232–247 656403 10.1016/0005-2728(78)90045-2

[B44] Flügge U.-I. Fischer K. Gross A. Sebald W. Lottspeich F. Eckerskorn C. (1989) The triose phosphate-3-phosphoglycerate-phosphate translocator from spinach chloroplasts: nucleotide sequence of a full-length cDNA clone and import of the in vitro synthesized precursor protein into chloroplasts. *EMBO J.* 8 39–46 2714257 10.1002/j.1460-2075.1989.tb03346.x PMC400770

[B45] Flügge U.-I. Häusler R. E. Ludewig F. Gierth M. (2011) The role of transporters in supplying energy to plant plastids. *J. Exp. Bot.* 62 2381–2392 10.1093/jxb/erq361 21511915

[B46] Fondy B. R. Geiger D. R. (1977) Sugar selectivity and other characteristics of phloem loading in *Beta vulgaris* L. *Plant Physiol.* 59 953–960 10.1104/pp.59.5.953 16659975 PMC543334

[B47] Fox S. R. Rawsthorne S. Hills M. J. (2001) Fatty acid synthesis in pea root plastids is inhibited by the action of long-chain acyl-coenzyme as on metabolite transporters. *Plant Physiol.* 126 1259–1265 10.1104/pp.126.3.1259 11457976 PMC116482

[B48] Furumoto T. Yamaguchi T. Ohshima-Ichie Y. Nakamura M. Tsuchida-Iwata Y. Shimamura M. (2011) A plastidial sodium-dependent pyruvate transporter. *Nature* 476 472–475 10.1038/nature10250 21866161

[B49] Gahrtz M. Stolz J. Sauer N. (1994) A phloem-specific sucrose-H+ symporter from *Plantago major* L. supports the model of apoplastic phloem loading*. Plant J.* 6 697–706 10.1046/j.1365-313X.1994.6050697.x 8000426

[B50] Getz H. P. (1991) Sucrose transport in tonoplast vesicles of red beet roots is linked to ATP hydrolysis. *Planta* 185 261–268 10.1007/BF00194069 24186350

[B51] Getz H. P. Klein M. (1995) Characteristics of sucrose transport and sucrose-induced H+ transport on the tonoplast of red beet (*Beta vulgaris* L.) storage tissue. *Plant Physiol.* 107 459–467 12228372 10.1104/pp.107.2.459 PMC157148

[B52] Giaquinta R. (1977a) Phloem loading of sucrose. pH dependency and selectivity*. Plant Physiol.* 59 750–755 10.1104/pp.59.4.750 PMC542486 16659931

[B53] Giaquinta R. (1977b) Possible role of pH gradient and membrane ATPase in the loading of sucrose into the sieve tubes. *Nature* 267 369–370 10.1038/267369a0

[B54] Giaquinta R. T. (1979) Sucrose translocation and storage in the sugar beet. *Plant Physiol.* 63 828–832 10.1104/pp.63.5.828 16660821 PMC542928

[B55] Godt D. Roitsch T. (2006) The developmental and organ specific expression of sucrose cleaving enzymes in sugar beet suggests a transition between apoplasmic and symplasmic phloem unloading in the tap roots. *Plant Physiol. Biochem.* 44 656–665 10.1016/j.plaphy.2006.09.019 17095237

[B56] Gómez E. Royo J. Guo Y. Thompson R. Hueros G. (2002) Establishment of cereal endosperm expression domains: identification and properties of a maize transfer cell-specific transcription factor, ZmMRP-1. *Plant Cell* 14 599–610 11910007 10.1105/tpc.010365 PMC150582

[B57] Gottwald J. R. Krysan P. J. Young J. C. Evert R. F. Sussman M. R. (2000) Genetic evidence for the in planta role of phloem-specific plasma membrane sucrose transporters. *Proc. Natl. Acad. Sci.* 97 13979–13984 10.1073/pnas.250473797 11087840 PMC17686

[B58] Gould N. Thorpe M. R. Pritchard J. Christeller J. T. Williams L. E. Roeb G. (2012) AtSUC2 has a role for sucrose retrieval along the phloem pathway: evidence from carbon-11 tracer studies. *Plant Sci.* 188–189 97–101 10.1016/j.plantsci.2011.12.018 22525249

[B59] Grof C. P. L. Campbell J. A. (2001) Sugarcane sucrose metabolism: scope for molecular manipulation. *Aust. J. Plant Physiol.* 28 1–12

[B60] Hafke J. B. van Amerongen J.-K. Kelling F. Furch A. C. U. Gaupels F van Bel A. J. E. (2005) Thermodynamic battle for photosynthate acquisition between sieve tubes and adjoining parenchyma in transport phloem. *Plant Physiol.* 138 1527–1537 10.1104/pp.104.058511 15980202 PMC1176423

[B61] Hajirezaei M.-R. Takahata Y. Trethewey R. N. Willmitzer L. Sonnewald U. (2000) Impact of elevated cytosolic and apoplastic invertase activity on carbon metabolism during potato tuber development. *J. Exp. Bot.* 51 439–445 10.1093/jexbot/51.suppl_1.439 10938852

[B62] Häusler R. E. Schlieben N. H. Nicolay P. Fischer K. Fischer K. L Flügge U.-I. (2000) Control of carbon partitioning and photosynthesis by the triose phosphate/phosphate translocator in transgenic tobacco plants (*Nicotiana tabacum* L.) I. *Comparative physiological analysis of tobacco plants with antisense repression and overexpression of the triose phosphate/phosphate translocator. Planta* 210 371–382 10.1007/PL00008145 10750894

[B63] Häusler R. E. Schlieben N. H. Schulz B Flügge U.-I. (1998) Compensation of decreased triose phosphate/phosphate translocator activity by accelerated starch turnover and glucose transport in transgenic tobacco. *Planta* 204 366–376 9530880 10.1007/s004250050268

[B64] He Y.-Q. Wu Y. (2009) Oil body biogenesis during *Brassica napus* embryogenesis. *J. Integr. Plant Biol.* 51 792–799 10.1111/j.1744-7909.2009.00851.x 19686376

[B65] Heineke D. Kruse A. Flügge U.-I. Frommer W. B. Riesmeier J. W. Willmitzer L. (1994) Effect of antisense repression of the chloroplast triose-phosphate translocator on photosynthetic metabolism in transgenic potato plants. *Planta* 193 174–180

[B66] Heineke D. Sonnewald U. Büssis D. Günter G. Leidreiter K. Wilke I. (1992) Apoplastic expression of yeast-derived invertase in potato: effects on photosynthesis, leaf solute composition, water relations, and tuber composition. *Plant Physiol.* 100 301–308 16652961 10.1104/pp.100.1.301 PMC1075552

[B67] Herman E. M. Larkins B. A. (1999) Protein storage bodies and vacuoles. *Plant Cell* 11 601–613 10213781 10.1105/tpc.11.4.601 PMC144198

[B68] Hsieh K Huang A. H. C. (2004) Endoplasmic reticulum, oleosins, and oils in seeds and tapetum cells. *Plant Physiol.* 136 3427–3434 10.1104/pp.104.051060 15542496 PMC527141

[B69] Imlau A. Truernit E. Sauer N. (1999) Cell-to-cell and long-distance trafficking of the green fluorescent protein in the phloem and symplastic unloading of the protein into sink tissues. *Plant Cell* 11 309–322 10072393 10.1105/tpc.11.3.309 PMC144181

[B70] James M. G. Denyer K. Myers A. M. (2003) Starch synthesis in the cereal endosperm. *Curr. Opin. Plant Biol.* 6 215–222 10.1016/S1369-5266(03)00042-6 12753970

[B71] Jonik C. Sonnewald U. Hajirezaei M. R. Flügge U.-I. Ludewig F. (2012) Simultaneous boosting of source and sink capacities doubles tuber starch yield of potato plants. *Plant Biotechnol. J.* 10 1088–1098 10.1111/j.1467-7652.2012.00736.x 22931170

[B72] Kammerer B. Fischer K. Hilpert B. Schubert S. Gutensohn M. Weber A. (1998) Molecular characterization of a carbon transporter in plastids from heterotrophic tissues: the glucose 6-phosphate/phosphate antiporter. *Plant Cell* 10 105–117 9477574 10.1105/tpc.10.1.105 PMC143937

[B73] Kampfenkel K. Möhlmann T. Batz O. van Montagu M. Inzé D. Neuhaus H. E. (1995) Molecular characterization of an *Arabidopsis thaliana* cDNA encoding a novel putative adenylate translocator of higher plants. *FEBS Lett.* 374 351–355 7589569 10.1016/0014-5793(95)01143-3

[B74] Kempers R. Ammerlaan A van Bel A. J. E. (1998) Symplasmic constriction and ultrastructural feature of the sieve element/companion cell complex in the transport phloem of apoplasmically and symplasmically phloem-loading species. *Plant Physiol.* 116 271–278 10.1104/pp.116.1.271

[B75] Kennedy E. P. (1961) Biosynthesis of complex lipids. *Fed. Proc.* 20 934–940 14455159

[B76] Kim S. Yamaoka Y. Ono H. Kim H. Shim D. Maeshima M. (2013) AtABCA9 transporter supplies fatty acids for lipid synthesis to the endoplasmic reticulum. *Proc. Natl. Acad. Sci. U.S.A.* 110 773–778 10.1073/pnas.1214159110 23269834 PMC3545803

[B77] Kirchberger S. Leroch M. Huynen M. A. Wahl M. Neuhaus H. E. Tjaden J. (2007) Molecular and biochemical analysis of the plastidic ADP-glucose transporter (ZmBT1) from *Zea mays*. *J. Biol. Chem.* 282 22481–22491 10.1074/jbc.M702484200 17562699

[B78] Klotz K. L. Finger F. L. (2002) Contribution of invertase and sucrose synthase isoforms to sucrose catabolism in developing sugarbeet roots. *J. Sugar Beet Res.* 39 1–24 10.5274/jsbr.39.1.1

[B79] Komor E. Rotter M. Tanner W. (1977) A proton-cotransport system in a higher plant: sucrose transport in *Ricinus communis*. *Plant Sci. Lett.* 9 153–162 10.1016/0304-4211(77)90093-1

[B80] Krishnan S. Dayanandan P. (2003) Structural and histochemical studies on grain-filling in the caryopsis of rice (*Oryza sativa* L.) *. J. Biosci. * 28 455–469 10.1007/BF02705120 12799492

[B81] Kühn C Grof C. P. L. (2010) Sucrose transporters of higher plants. *Curr. Opin. Plant Biol.* 13 288–298 10.1016/j.pbi.2010.02.001 20303321

[B82] Kühn C. Quick W. P. Schulz A. Sonnewald U. Frommer W. B. (1996) Companion cell-specific inhibition of the potato sucrose transporter SUT1. *Plant Cell Environ.* 19 1115–1123 10.1111/j.1365-3040.1996.tb00426.x

[B83] Leggewie G. Kolbe A. Lemoine R. Roessner U. Lytovchenko A. Zuther E. (2003) Overexpression of the sucrose transporter SoSUT1 in potato results in alterations in leaf carbon partitioning and in tuber metabolism but has little impact on tuber morphology. *Planta* 217 158–167 12721860 10.1007/s00425-003-0975-x

[B84] Leidreiter K. Heineke D. Heldt H. W. Müller-Röber B. Sonnewald U. Willmitzer L. (1995) Leaf-specific antisense inhibition of starch biosynthesis in transgenic potato plants leads to an increase in photoassimilate export from source leaves during the light period. *Plant Cell Physiol.* 36 615–624

[B85] Li H. M. Culligan K. Dixon R. A. Chory J. (1995) CUE1: a mesophyll cell-specific positive regulator of light-controlled gene expression in *Arabidopsis*. *Plant Cell* 7 1599–1610 12242356 10.1105/tpc.7.10.1599 PMC161018

[B86] Li N. Zhang S. Zhao Y. Li B. Zhang J. (2011) Over-expression of AGPase genes enhances seed weight and starch content in transgenic maize. *Planta* 233 241–250 10.1007/s00425-010-1296-5 20978801

[B87] Li-Beisson Y. Shorrosh B. Beisson F. Andresson M. X. Arondel V. Bates P. D. (2013) Acyl-lipid metabolism. *Arabidopsis Book* 11: e0161 10.1199/tab.0161 PMC3563272 23505340

[B88] Lohaus G. Burba M. Heldt H. W. (1994) Comparison of the contents of sucrose and amino acids in the leaves, phloem sap and taproots of high and low sugar-producing hybrids of sugar beet (*Beta vulgaris* L.) *J. Exp. Bot.* 45 1097–1101 10.1093/jxb/45.8.1097

[B89] Lohaus G. Winter H. Riens B. Heldt H. W. (1995) Further studies on the phloem loading process in leaves of barley and spinach. The comparison of metabolite concentrations in the apoplastic compartment with those in the cytosolic compartment and in sieve tubes. *Bot. Acta* 108 270–275

[B90] Long S. P. Zhu X.-G. Naidu S. L. Ort D. R. (2006) Can improvement in photosynthesis increase crop yields? *Plant Cell Environ.* 29 315–330 10.1111/j.1365-3040.2005.01493.x 17080588

[B91] Makino A. (2011) Photosynthesis, grain yield, and nitrogen utilization in rice and wheat. *Plant Physiol.* 155 125–129 10.1104/pp.110.165076 20959423 PMC3014227

[B92] Manning K. Davies C. Bowen H. C. White P. J. (2001) Functional characterization of two ripening-related sucrose transporters from grape berries. *Ann. Bot.* 87 125–129 10.1006/anbo.2000.1316

[B93] Martinoia E. Massonneau A. Frangne N. (2000) Transport processes of solutes across the vacuolar membrane of higher plants. *Plant Cell Physiol.* 41 1175–1186 10.1093/pcp/pcd059 11092901

[B94] McCormick A. J. Cramer M. D. Watt D. A. (2006) Sink strength regulates photosynthesis in sugarcane. *New Phytol.* 171 759–770 10.1111/j.1469-8137.2006.01785.x 16918547

[B95] McCormick A. J. Cramer M. D. Watt D. A. (2008) Changes in photosynthetic rates and gene expression of leaves during a source–sink perturbation in sugarcane. *Ann. Bot.* 101 89–102 10.1093/aob/mcm258 17942591 PMC2701831

[B96] McCouch S. (2004) Diversifying selection in plant breeding. *PLoS Biol. * 2: e347 10.1371/journal.pbio.0020347 PMC521731 15486582

[B97] McCurdy D. W. Dibley S. Cahyanegara R. Martin A. Patrick J. W. (2010) Functional characterization and RNAi-mediated suppression reveals roles for hexose transporters in sugar accumulation by tomato fruit. *Mol. Plant* 3 1049–1063 10.1093/mp/ssq050 20833733

[B98] Melkus G. Rolletschek H. Radchuk R. Fuchs J. Rutten T. Wobus U. (2009) The metabolic role of the legume endosperm: a noninvasive imaging study. *Plant Physiol.* 151 1139–1154 10.1104/pp.109.143974 19748915 PMC2773074

[B99] Meyer S. Lauterbach C. Niedermeier M. Barth I. Sjolund R. D. Sauer N. (2004) Wounding enhances expression of AtSUC3, a sucrose transporter from *Arabidopsis* sieve elements and sink tissues. *Plant Physiol.* 134 684–693 10.1104/pp.103.033399 14739351 PMC344544

[B100] Meyer S. Melzer M. Truernit E. Hümmer C. Besenbeck R. Stadler R. (2000) AtSUC3, a gene encoding a new *Arabidopsis* sucrose transporter, is expressed in cells adjacent to the vascular tissue and in a carpel cell layer. *Plant J.* 24 869–882 11135120 10.1046/j.1365-313x.2000.00934.x

[B101] Minchin P. E. H. Ryan K. G. Thorpe M. R. (1984) Further evidence of apoplastic unloading into the stem of bean: identification of the phloem buffering pool. *J. Exp. Bot.* 35 1744–1753 10.1093/jxb/35.12.1744

[B102] Minchin P. E. H. Thorpe M. R. (1987) Measurement of unloading and reloading of photo-assimilate within the stem of bean. *J. Exp. Bot.* 38 211–220 10.1093/jxb/38.2.211

[B103] Miranda M. Borisjuk L. Tewes A. Heim U. Sauer N. Wobus U. (2001) Amino acid permeases in developing seeds of *Vicia faba* L.: expression precedes storage protein synthesis and is regulated by amino acid supply. *Plant J*. 28 61–72 10.1046/j.1365-313X.2001.01129.x 11696187

[B104] Möhlmann T. Tjaden J. Henrichs G. Quick W. P. Häusler R. Neuhaus H. E. (1997) ADP-glucose drives starch synthesis in isolated maize endosperm amyloplasts: characterization of starch synthesis and transport properties across the amyloplast envelope. *Biochem. J.* 324 503–509 9182710 10.1042/bj3240503 PMC1218458

[B105] Münch E. (1930) *Die Stoffbewegungen in der Pflanze*. Jena: Verlag Gustav Fischer 234

[B106] Nguyen-Quoc B. Foyer C. H. (2001) A role for ‘futile cycles’ involving invertase and sucrose synthase in sucrose metabolism of tomato fruit. *J. Exp. Bot.* 52 881–889 10.1093/jexbot/52.358.881 11432905

[B107] Niittylä T. Messerli G. Trevisan M. Chen J. Smith A. M. Zeeman S. C. (2004) A previously unknown maltose transporter essential for starch degradation in leaves. *Science* 303 87–89 10.1126/science.1091811 14704427

[B108] Patrick J. W. Offler C. E. (2001) Compartmentation of transport and transfer events in developing seeds. *J. Exp. Bot.* 52 551–564 10.1093/jexbot/52.356.551 11373304

[B109] Paul M. J. Foyer C. H. (2001) Sink regulation of photosynthesis. *J. Exp. Bot.* 52 1383–1400 10.1093/jexbot/52.360.1383 11457898

[B110] Paul M. J. Pellny T. K. (2003) Carbon metabolite feedback regulation of leaf photosynthesis and development. *J. Exp. Bot.* 54 539–547 10.1093/jxb/erg052 12508065

[B111] Payyavula R. S. Tay K. H. C. Tsai C.-J. Harding S. A. (2011) The sucrose transporter family in *Populus*: the importance of a tonoplast PtaSUT4 to biomass and carbon partitioning. *Plant J.* 65 757–770 10.1111/j.1365-313X.2010.04463.x 21261761

[B112] Periappuram C. Steinhauer L. Barton D. L. Taylor D. C. Chatson B. Zou J. (2000) The plastidic phosphoglucomutase from *Arabidopsis*. A reversible enzyme reaction with an important role in metabolic control.* Plant Physiol.* 122 1193–1199 10.1104/pp.122.4.1193 PMC58954 10759515

[B113] Petreikov M. Yeselson L. Shen S. Levin I. Schaffer A. A. Efrati A. (2009) Carbohydrate balance and accumulation during development of near-isogenic tomato lines differing in the AGPase-L1 allele. *J. Am. Soc. Hortic. Sci.* 134 134–140

[B114] Pommerrenig B. Popko J. Heilmann M. Schulmeister S. Dietel K. Schmitt B. (2013) SUCROSE TRANSPORTER 5 supplies *Arabidopsis* embryos with biotin and affects triacylglycerol accumulation. *Plant J.* 73 392–404 10.1111/tpj.12037 23031218 PMC3787789

[B115] Poschet G. Hannich B. Raab S. Jungkunz I. Klemens P. A. W. Krüger S. (2011) A novel *Arabidopsis* vacuolar glucose exporter is involved in cellular sugar homeostasis and affects the composition of seed storage compounds. *Plant Physiol.* 157 1664–1676 21984725 10.1104/pp.111.186825 PMC3327193

[B116] Rae A. L. Jackson M. A. Nguyen C. H. Bonnett G. D. (2009) Functional specialization of vacuoles in sugarcane leaf and stem. *Tropical Plant Biol.* 2 13–22 10.1007/s12042-008-9019-9

[B117] Rae A. L. Perroux J. M Grof C. P. L. (2005) Sucrose partitioning between vascular bundles and storage parenchyma in the sugarcane stem: a potential role for the ShSUT1 sucrose transporter. *Planta* 220 817–825 10.1007/s00425-004-1399-y 15517352

[B118] Ramaiah M. Jain A. Baldwin J. C. Karthikeyan A. S. Raghothama K. G. (2011) Characterization of the phosphate starvation-induced glycerol-3-phosphate permease gene family in *Arabidopsis*. *Plant Physiol.* 157 279–291 10.1104/pp.111.178541 21788361 PMC3165876

[B119] Rawsthorne S. (2002) Carbon flux and fatty acid synthesis in plants. *Prog. Lipid Res.* 41 182–196 10.1016/S0163-7827(01)00023-6 11755683

[B120] Reidel E. J. Turgeon R. Cheng L. (2008) A maltose transporter from apple is expressed in source and sink tissues and complements the *Arabidopsis* maltose export-defective mutant. *Plant Cell Physiol.* 49 1607–1613 10.1093/pcp/pcn134 18776201

[B121] Reinders A. Sivitz A. B. Starker C. G. Gantt J. S. Ward J. M. (2008) Functional analysis of LjSUT4, a vacuolar sucrose transporter from *Lotus japonicus*. *Plant Mol. Biol.* 68 289–299 10.1007/s11103-008-9370-0 18618272

[B122] Reinders A. Sivitz A. B. Ward J. M. (2012) Evolution of plant sucrose uptake transporters. *Front. Plant Sci. * 3: 22 10.3389/fpls.2012.00022 PMC3355574 22639641

[B123] Reiser J. Linka N. Lemke L. Jeblick W. Neuhaus H. E. (2004) Molecular physiological analysis of the two plastidic ATP/ADP transporters from *Arabidopsis*. *Plant Physiol.* 136 3524–3536 10.1104/pp.104.049502 15516503 PMC527152

[B124] Renné P. Dressen U. Hebbeker U. Hille D. Flügge U.-I. Westhoff P. (2003) The *Arabidopsis *mutant dct is deficient in the plastidic glutamate/malate translocator DiT2. *Plant J.* 35 316–331 12887583 10.1046/j.1365-313x.2003.01806.x

[B125] Rennie E. A. Turgeon R. (2009) A comprehensive picture of phloem loading strategies. *Proc. Natl. Acad. Sci. U.S.A.* 106 14162–14167 10.1073/pnas.0902279106 19666555 PMC2729037

[B126] Riesmeier J. W. Flügge U.-I. Schulz B. Heineke D. Heldt H.-W. Willmitzer L. (1993a) Antisense repression of the chloroplast triose phosphate translocator affects carbon partitioning in transgenic potato plants. *Proc. Natl. Acad. Sci. U.S.A.* 90 6160–6164 11607409 10.1073/pnas.90.13.6160 PMC46887

[B127] Riesmeier J. W. Hirner B. Frommer W. B. (1993b) Potato sucrose transporter expression in minor veins indicates a role in phloem loading. *Plant Cell* 5 1591–1598 8312741 10.1105/tpc.5.11.1591 PMC160388

[B128] Riesmeier J. W. Willmitzer L. Frommer W. B. (1992) Isolation and characterization of a sucrose carrier cDNA from spinach by functional expression in yeast. *EMBO J.* 11 4705–4713 1464305 10.1002/j.1460-2075.1992.tb05575.x PMC556945

[B129] Riesmeier J. W. Willmitzer L. Frommer W. B. (1994) Evidence for an essential role of the sucrose transporter in phloem loading and assimilate partitioning. *EMBO J.* 13 1–7 8306952 10.1002/j.1460-2075.1994.tb06229.x PMC394773

[B130] Robinson N. L. Hewitt J. D. Bennett A. B. (1988) Sink metabolism in tomato fruit. I. Developmental changes in carbohydrate metabolizing enzymes. *Plant Physiol.* 87 727–730 10.1104/pp.87.3.727 PMC1054828 16666215

[B131] Rodrïguez-Falcón M. Bou J. Prat S. (2006) Seasonal control of tuberization in potato: conserved elements with the flowering response. *Annu. Rev. Plant Biol.* 57 151–180 16669759 10.1146/annurev.arplant.57.032905.105224

[B132] Rolletschek H. Hosein F. Miranda M. Heim U. Götz K.-P. Schlereth A. (2005) Ectopic expression of an amino acid transporter (VfAAP1) in seeds of *Vicia narbonensis* and pea increases storage proteins. *Plant Physiol.* 137 1236–1249 15793070 10.1104/pp.104.056523 PMC1088317

[B133] Rolletschek H. Nguyen T. H. Häusler R. E. Rutten T. Göbel C. Feussner I. (2007) Antisense inhibition of the plastidial glucose-6-phosphate/phosphate translocator in *Vicia* seeds shifts cellular differentiation and promotes protein storage. *Plant J.* 51 468–484 17587237 10.1111/j.1365-313X.2007.03155.x

[B134] Rosche E. Blackmore D. Tegeder M. Richardson T. Schroeder H. Higgins T. J. V., et al. (2002) Seed-specific overexpression of a potato sucrose transporter increases sucrose uptake and growth rates of developing pea cotyledons. *Plant J.* 31 165–175 10.1046/j.1365-313X.2002.01282.x 12000453

[B135] Ruan Y.-L. Patrick J. W. (1995) The cellular pathway of postphloem sugar transport in developing tomato fruit. *Planta* 196 434–444 10.1007/BF00203641

[B136] Ruan Y.-L. Patrick J. W. Brady C. (1997) Protoplast hexose carrier activity is a determinate of genotypic difference in hexose storage in tomato fruit. *Plant Cell Environ.* 20 341–349 10.1046/j.1365-3040.1997.d01-73.x

[B137] Saftner R. A. Daie J. Wyse R. E. (1983) Sucrose uptake and compartmentation in sugar beet taproot tissue. *Plant Physiol.* 72 1–6 10.1104/pp.72.1.1 16662941 PMC1066159

[B138] Sauer N. (2007) Molecular physiology of higher plant sucrose transporters. *FEBS Lett.* 581 2309–2317 10.1016/j.febslet.2007.03.048 17434165

[B139] Sauer N. Stolz J. (1994) SUC1 and SUC2: two sucrose transporters from *Arabidopsis* *thaliana*; expression and characterization in baker’s yeast and identification of the histidine-tagged protein. *Plant J.* 6 67–77 10.1046/j.1365-313X.1994.6010067.x 7920705

[B140] Schaffer A. A. Petreikov M. (1997) Sucrose-to-starch metabolism in tomato fruit undergoing transient starch accumulation. *Plant Physiol.* 113 739–746 12223639 10.1104/pp.113.3.739 PMC158191

[B141] Schattat M. Barton K. Baudisch B. Klösgen R. B. Mathur J. (2011) Plastid stromule branching coincides with contiguous endoplasmic reticulum dynamics. *Plant Physiol.* 155 1667–1677 10.1104/pp.110.170480 21273446 PMC3091094

[B142] Schmitt B. Stadler R. Sauer N. (2008) Immunolocalization of solanaceous SUT1 proteins in companion cells and xylem parenchyma: new perspectives for phloem loading and transport. *Plant Physiol.* 148 187–199 10.1104/pp.108.120410 18614713 PMC2528081

[B143] Schmitz J. Schöttler M. A. Krüger S. Geimer S. Schneider A. Kleine T. (2012) Defects in leaf carbohydrate metabolism compromise acclimation to high light and lead to a high chlorophyll fluorescence phenotype in *Arabidopsis thaliana*. *BMC Plant Biol. * 12: 8 10.1186/1471-2229-12-8 PMC3353854 22248311

[B144] Schneider A. Häusler R. E. Kolukisaoglu ü Kunze R. van der Graaff E. Schwacke R. (2002) An *Arabidopsis thaliana* knock-out of the chloroplast triose phosphate/phosphate transloctor is severely compromised only when starch synthesis, but not starch mobilization is abolished. *Plant J*. 32 685–699 12472685 10.1046/j.1365-313x.2002.01460.x

[B145] Schneider S. Hulpke S. Schulz A. Yaron I. Höll J. Imlau A. (2012) Vacuoles release sucrose via tonoplast-localised SUC4-type transporters. *Plant Biol.* 14 325–336 10.1111/j.1438-8677.2011.00506.x 21972845

[B146] Schneidereit A. Scholz-Starke J Büttner M. (2003) Functional characterization and expression analyses of the glucose-specific AtSTP9 monosaccharide transporter in pollen of *Arabidopsis*. *Plant Physiol.* 133 182–190 12970485 10.1104/pp.103.026674 PMC196596

[B147] Schulz A. Beyhl D. Marten I. Wormit A. Neuhaus E. Poschet G. (2011) Proton-driven sucrose symport and antiport are provided by the vacuolar transporters SUC4 and TMT1/2. *Plant J.* 68 129–136 10.1111/j.1365-313X.2011.04672.x 21668536

[B148] Shannon J. C. Pein F. M. Cao H. P. Liu K. C. (1998) Brittle-1, an adenylate translocator, facilitates transfer of extraplastidial synthesized ADP-glucose into amyloplasts of maize endosperm. *Plant Physiol.* 117 1235–1252 10.1104/pp.117.4.1235 9701580 PMC34888

[B149] Shockey J. M. Fulda M. S. Browse J. A. (2002) *Arabidopsis* contains nine long-chain acyl-coenzyme A synthetase genes that participate in fatty acid and glycerolipid metabolism. *Plant Physiol.* 129 1710–1722 10.1104/pp.003269 12177484 PMC166759

[B150] Sivitz A. B. Reinders A. Johnson M. E. Krentz A. D. Grof C. P. L. Perroux J. M. (2007) *Arabidopsis* sucrose transporter SUC9. High-affinity transport activity, intragenic control of expression, and early flowering mutant phenotype*. Plant Physiol.* 143 188–198 10.1104/pp.106.089003 PMC1761979 17098854

[B151] Slewinski T. L. Garg A. Johal G. S. Braun D. M. (2010) Maize SUT1 functions in phloem loading. *Plant Signal. Behav.* 5 687–690 10.4161/psb.5.6.11575 20404497 PMC3001560

[B152] Slewinski T. L. Meeley R. Braun D. M. (2009) Sucrose transporter1 functions in phloem loading in maize leaves. *J. Exp. Bot.* 60 881–892 10.1093/jxb/ern335 19181865 PMC2652052

[B153] Smidansky E. D. Clancy M. Meyer F. D. Lanning S. P. Blake N. K. Talbert L. E. (2002) Enhanced ADP-glucose pyrophosphorylase activity in wheat endosperm increases seed yield. *Proc. Natl. Acad. Sci. U.S.A.* 99 1724–1729 10.1073/pnas.022635299 11830676 PMC122258

[B154] Somerville S. C. Ogren W. L. (1983) An *Arabidopsis thaliana* mutant defective in chloroplast dicarboxylate transport. *Proc. Natl. Acad. Sci. U.S.A.* 80 1290–1294 10.1073/pnas.80.5.1290 16593285 PMC393581

[B155] Somerville S. C. Somerville C. R. (1985) A mutant of *Arabidopsis* deficient in chloroplast dicarboxylate transport is missing an envelope protein. *Plant Sci. Lett.* 37 217–220 10.1016/0304-4211(85)90007-0

[B156] Sonnewald U. (2011) SWEETS – the missing sugar efflux carriers. *Front. Plant Sci.* 2: 7 10.3389/fpls.2011.00007 PMC3355597 22639574

[B157] Sonnewald U. Brauer M. von Schaewen A. Stitt M. Willmitzer L. (1991) Transgenic tobacco plants expressing yeast-derived invertase in either the cytosol, vacuole or apoplast: a powerful tool for studying sucrose metabolism and sink/source interactions. *Plant J.* 1 95–106 10.1111/j.1365-313X.1991.00095.x 1844880

[B158] Sonnewald U. Hajirezaei M.-R. Kossmann J. Heyer A. Trethewey R. N. Willmitzer L. (1997) Increased potato tuber size resulting from apoplastic expression of a yeast invertase. *Nat. Biotechnol.* 15 794–797 10.1038/nbt0897-794 9255797

[B159] Srivastava A. C. Ganesan S. Ismail I. O. Ayre B. G. (2008) Functional characterization of the *Arabidopsis* AtSUC2 sucrose/H+ symporter by tissue-specific complementation reveals an essential role in phloem loading but not in long-distance transport. *Plant Physiol.* 148 200–211 10.1104/pp.108.124776 18650401 PMC2528097

[B160] Stadler R. Brandner J. Schulz A. Gahrtz M. Sauer N. (1995) Phloem loading by the PmSUC2 sucrose carrier from *Plantago major* occurs into companion cells. *Plant Cell* 7 1545–1554 10.1105/tpc.7.10.1545 12242355 PMC161007

[B161] Stadler R. Sauer N. (1996) The *Arabidopsis thaliana* AtSUC2 gene is specifically expressed in companion cells. *Bot. Acta* 109 299–306 10.1104/pp.008037

[B162] Stadler R. Wright K. M. Lauterbach C. Amon G. Gahrtz M. Feuerstein A. (2005a) Expression of GFP-fusions in *Arabidopsis* companion cells reveals non-specific protein trafficking into sieve elements and identifies a novel post-phloem domain in roots. *Plant J.* 41 319–331 10.1111/j.1365-313X.2004.02298.x 15634207

[B163] Stadler R. Lauterbach C. Sauer N. (2005b) Cell-to-cell movement of green fluorescent protein reveals post-phloem transport in the outer integument and identifies symplastic domains in *Arabidopsis* seeds and embryos. *Plant Physiol.* 139 701–712 10.1104/pp.105.065607 16169962 PMC1255989

[B164] Stettler M. Eicke S. Mettler T. Messerli G. Hörtensteiner S. Zeeman S. (2009) Blocking the metabolism of starch breakdown products in *Arabidopsis* leaves triggers chloroplast degradation. *Mol. Plant* 2 1233–1246 10.1093/mp/ssp093 19946617 PMC2782796

[B165] Stitt M. (1991) Rising CO2 levels and their potential significance for carbon flow in photosynthetic cells. *Plant Cell. Environ.* 14 741–762 10.1111/j.1365-3040.1991.tb01440.x

[B166] Streatfield S. J. Weber A. Kinsman E. A. Häusler R. E. Li J. Post-Beittenmiller D. (1999) The phosphoenolpyruvate/phosphate translocator is required for phenolic metabolism, palisade cell development, and plastid-dependent nuclear gene expression. *Plant Cell* 11 1609–1621 10488230 10.1105/tpc.11.9.1609 PMC144315

[B167] Sullivan T. D. Kaneko Y. (1995) The maize brittle-1 gene encodes amyloplast membrane polypeptides. *Planta* 196 477–484 10.1007/BF00203647 7647682

[B168] Tegeder M. Wang X.-D. Frommer W. B. Offler C. E. Patrick J. W. (1999) Sucrose transport into developing seed of *Pisum sativum* L. *Plant J.* 18 151–161 10.1046/j.1365-313X.1999.00439.x 10363367

[B169] Thorbjörnsen T. Villand P. Denyer K. Olsen O.-A. Smith A. M. (1996) Distinct isoforms of ADPglucose pyrophosphorylase occur inside and outside the amyloplasts in barley endosperm. *Plant J.* 10 243–250 10.1046/j.1365-313X.1996.10020243.x

[B170] Tjaden J. Möhlmann T. Kampfenkel K. Henrichs G. Neuhaus H. E. (1998) Altered plastidic ATP/ADP-translocator activity influences potato (*Solanum tuberosum* L.) tuber morphology, yield and composition of tuber starch. *Plant J.* 16 531–540 10.1046/j.1365-313x.1998.00317.x

[B171] Trentmann O. Jung B. Neuhaus H. E. Haferkamp I. (2008) Non-mitochondrial ATP/ADP transporters accept phosphate as third substrate. *J. Biol. Chem.* 283 36486–36493 10.1074/jbc.M806903200 19001371 PMC2606016

[B172] Truernit E. Sauer N. (1995) The promoter of the *Arabidopsis thaliana* sucrose-H+ symporter gene directs expression of β-glucuronidase to the phloem: evidence for phloem loading and unloading by SUC2. *Planta* 196 564–570 10.1007/BF00203657 7647685

[B173] Turgeon R. (1989) The sink-source transition in leaves. *Annu. Rev. Plant Physiol. Plant Mol. Biol.* 40 119–138 10.1146/annurev.pp.40.060189.001003

[B174] Uys L. Botha F. C. Hofmeyr J.-H. S. Rohwer J. M. (2007) Kinetic model of sucrose accumulation in maturing sugarcane culm tissue. *Phytochemistry* 68 2375–2392 10.1016/j.phytochem.2007.04.023 17555779

[B175] van Dongen J. T. Ammerlaan A. M. H. Wouterlood M. van Aelst A. C. Borstlap A. C. (2003) Structure of the developing pea seed coat and the post-phloem transport pathway of nutrients. *Ann. Bot.* 91 729–737 10.1093/aob/mcg066 12714370 PMC4242349

[B176] Vigeolas H. Möhlmann T. Martini N. Neuhaus H. E. Geigenberger P. (2004) Embryo-specific reduction of ADP-Glc pyrophosphorylase leads to an inhibition of starch synthesis and a delay in oil accumulation in developing seeds of oilseed rape. *Plant Physiol.* 136 2676–2686 10.1104/pp.104.046854 15333758 PMC523332

[B177] Viola R. Roberts A. G. Haupt S. Gazzani S. Hancock R. D. Marmiroli N. (2001) Tuberization in potato involves a switch from apoplastic to symplastic phloem unloading. *Plant Cell* 13 385–398 11226192 10.1105/tpc.13.2.385 PMC102249

[B178] Vitale A. Ceriotti A. (2004) Protein quality control mechanisms and protein storage in the endoplasmic reticulum. A conflict of interests?* Plant Physiol.* 136 3420–3426 10.1104/pp.104.050351 PMC527140 15542495

[B179] Voll L. Häusler R. E. Hecker R. Weber A. Weissenböck G. Fiene G. (2003) The phenotype of the *Arabidopsis* cue1 mutant is not simply caused by a general restriction of the shikimate pathway. *Plant J.* 36 301–317 14617088 10.1046/j.1365-313x.2003.01889.x

[B180] von Schaewen A. Stitt M. Schmidt R. Sonnewald U. Willmitzer L. (1990) Expression of a yeast-derived invertase in the cell wall of tobacco and *Arabidopsis* plants leads to accumulation of carbohydrate and inhibition of photosynthesis and strongly influences growth and phenotype of transgenic tobacco plants. *EMBO J.* 9 3033–3044 2209536 10.1002/j.1460-2075.1990.tb07499.x PMC552027

[B181] Walters R. G. Ibrahim D. G. Horton P. Kruger N. J. (2004) A mutant of *Arabidopsis* lacking the triose-phosphate/phosphate translocator reveals metabolic regulation of starch breakdown in the light. *Plant Physiol.* 135 891–906 10.1104/pp.104.040469 15173568 PMC514124

[B182] Wang Z. Chen X. Wang J. Liu T. Liu Y. Zhao L. (2007) Increasing maize seed weight by enhancing the cytoplasmic ADP-glucose pyrophosphorylase activity in transgenic maize plants. *Plant Cell Tissue Organ Cult.* 88 83–92 10.1007/s11240-006-9173-4

[B183] Weber A. Servaites J. C. Geiger D. R. Kofler H. Hille D. Gröner F. (2000) Identification, purification, and molecular cloning of a putative plastidic glucose translocator. *Plant Cell* 12 787–802 10810150 10.1105/tpc.12.5.787 PMC139927

[B184] Weber H. Borisjuk L. Heim U. Sauer N. Wobus U. (1997) A role for sugar transporters during seed development: molecular characterization of a hexose and a sucrose carrier in fava bean seeds. *Plant Cell* 9 895–908 10.1105/tpc.9.6.895 9212465 PMC156966

[B185] Weise A. Barker L. Kühn C. Lalonde S. Buschmann H. Frommer W. B. (2000) A new subfamily of sucrose transporters, SUT4, with low affinity/high capacity localized in enucleate sieve elements of plants. *Plant Cell* 12 1345–1355 10.1105/tpc.12.8.1345 10948254 PMC149107

[B186] Weise S. E. Weber A. P. M. Sharkey T. D. (2004) Maltose is the major form of carbon exported from the chloroplast at night. *Planta* 218 474–482 10.1007/s00425-003-1128-y 14566561

[B187] Werner D. Gerlitz N. Stadler R. (2011) A dual switch in phloem unloading during ovule development in *Arabidopsis*. *Protoplasma* 248 225–235 10.1007/s00709-010-0223-8 21153670

[B188] Weschke W. Panitz R. Sauer N. Wang Q. Neubohn B. Weber H. (2000) Sucrose transport into barley seeds: molecular characterization of two transporters and implications for seed development and starch accumulation. *Plant J.* 21 455–467 10.1046/j.1365-313x.2000.00695.x 10758497

[B189] Whittaker A. Botha F. C. (1997) Carbon partitioning during sucrose accumulation in sugarcane intermodal tissue. *Plant Physiol.* 115 1651–1659 10.1104/pp.115.4.1651 12223886 PMC158631

[B190] Wingenter K. Schulz A. Wormit A. Wic S. Trentmann O. Hoermiller I. I. (2010) Increased activity of the vacuolar monosaccharide transporter TMT1 alters cellular sugar partitioning, sugar signaling, and seed yield in *Arabidopsis*. *Plant Physiol.* 154 665–677 10.1104/pp.110.162040 20709831 PMC2949046

[B191] Wingenter K. Trentmann O. Winschuh I. Hörmiller I. I. Heyer A. G. Reinders J. (2011) A member of the mitogen-activated protein 3-kinase family is involved in the regulation of plant vacuolar glucose uptake. *Plant J.* 68 890–900 10.1111/j.1365-313X.2011.04739.x 21838775

[B192] Winter D. Vinegar B. Nahal H. Ammar R. Wilson G. V. Provart N. J. (2007) An ‘electronic fluorescent pictograph’ browser for exploring and analyzing large-scale biological data sets. *PLoS ONE *2:e718. 10.1371/journal.pone.0000718 PMC1934936 17684564

[B193] Wippel K. Sauer N. (2012) *Arabidopsis* SUC1 loads the phloem in suc2 mutants when expressed from the SUC2 promoter. *J. Exp. Bot.* 63 669–679 10.1093/jxb/err255 22021573 PMC3254675

[B194] Wormit A. Trentmann O. Feifer I. Lohr C. Tjaden J. Meyer S. (2006) Molecular identification and physiological characterization of a novel monosaccharide transporter from *Arabidopsis* involved in vacuolar sugar transport. *Plant Cell* 18 3476–3490 10.1105/tpc.106.047290 17158605 PMC1785410

[B195] Wyse R. (1979) Sucrose uptake by sugar beet tap root tissue. *Plant Physiol.* 64 837–841 10.1104/pp.64.5.837 16661065 PMC543374

[B196] Yamada K. Osakabe Y. Mizoi J. Nakashima K. Fujita Y. Shinozaki K. (2010) Functional analysis of an *Arabidopsis thaliana* abiotic-stress-inducible facilitated diffusion transporter for monosaccharides. *J. Biol. Chem.* 285 1138–1146 10.1074/jbc.M109.054288 19901034 PMC2801242

[B197] Yu T.-S. Kofler H. Häusler R. E. Hille D. Flügge U.-I. Zeeman S. C. (2001) The *Arabidopsis sex1* mutant is defective in the R1 protein, a general regulator of starch degradation in plants, and not in the chloroplast hexose transporter. *Plant Cell* 13 1907–1918 11487701 10.1105/TPC.010091 PMC139133

[B198] Zhang L. Häusler R. E. Greiten C. Hajirezaei M.-R. Haferkamp I. Neuhaus H. E. (2008) Overriding the co-limiting import of carbon and energy into tuber amyloplasts increases the starch content and yield of transgenic potato plants. *Plant Biotechnol. J.* 6 453–464 10.1111/j.1467-7652.2008.00332.x 18363632

[B199] Zhang X.-Y. Wang X.-L. Wang X.-F. Xia G.-H. Pan Q.-H. Fan R.-C. (2006) A shift of phloem unloading from symplasmic to apoplasmic pathway is involved in developmental onset of ripening in grape berry. *Plant Physiol.* 142 220–232 10.1104/pp.106.081430 16861573 PMC1557625

[B200] Zhou Y. Chan K. Wang T. L. Hedley C. L. Offler C. E. Patrick J. W. (2009) Intracellular sucrose communicates metabolic demand to sucrose transporters in developing pea cotyledons. *J. Exp. Bot.* 60 71–85 10.1093/jxb/ern254 18931350 PMC3071760

[B201] Zhou Y. Qu H. Dibley K. E. Offler C. E. Patrick J. W. (2007) A suite of sucrose transporters expressed in coats of developing legume seeds includes novel pH-independent facilitators. *Plant J.* 49 750–764 10.1111/j.1365-313X.2006.03000.x 17253986

[B202] Zhu Y. J. Komor E. Moore P. H. (1997) Sucrose accumulation in the sugarcane stem is regulated by the difference between the activities of soluble acid invertase and sucrose phosphate synthase. *Plant Physiol.* 115 609–616 12223829 10.1104/pp.115.2.609 PMC158521

